# The Receptor for Advanced Glycation End-Products (RAGE) Regulates Cell Adhesion Through Upregulation of ITGA8

**DOI:** 10.3390/cells14221805

**Published:** 2025-11-17

**Authors:** Swetha Thiyagarajan, Estelle Leclerc, Stefan W. Vetter

**Affiliations:** Department of Pharmaceutical Sciences, North Dakota State University, Fargo, ND 58105, USA; swetha.thiyaga@gmail.com (S.T.); estelle.leclerc@ndsu.edu (E.L.)

**Keywords:** RAGE, RAGE domain deletion, extracellular matrix adhesion, cell adhesion molecules

## Abstract

The Receptor for Advanced Glycation End-Products (RAGE) is a cell surface receptor of the immunoglobulin-like receptor superfamily. RAGE is a pattern-recognition, multi-ligand receptor that binds glycated proteins, specific non-glycated proteins, and nucleic acids. RAGE ligands are typically part of the group of damage-associated molecular patterns (DAMPs) or alarmins. As such, RAGE is a receptor for molecular products of cellular stress, abnormal metabolism, and inflammation. Activation of RAGE by its ligands leads to pro-inflammatory signaling, often resulting in persistent RAGE activation in various disease states. Consequently, RAGE has been investigated as a potential drug target in the treatment of diabetic complications, vascular disease, Alzheimer’s disease, and multiple types of cancer. An underexplored aspect of RAGE is its role in cell adhesion. Structural comparison of the extracellular domain of RAGE has revealed structural similarity to the activated leukocyte cell adhesion molecule (ALCAM). The present study reveals the role and mechanism of RAGE in regulating cell adhesion. We investigated the role of individual RAGE domains in cell adhesion to extracellular matrix proteins and the changes in protein expression resulting from RAGE upregulation. Key findings include that RAGE displays substrate-specific adhesion to extracellular matrix proteins, that the intracellular domain of RAGE is required for modulating cell spreading, and that regulation of ITGA8 depends on the cytoplasmic domain of RAGE.

## 1. Introduction

The Receptor for Advanced Glycation End-products (RAGE) is a type I cell surface protein and a member of the immunoglobulin Ig protein superfamily. The RAGE protein was initially identified as a receptor for Advanced Glycation End-products (AGE) and was named for this property [1,2]. The gene encoding RAGE is named AGER (advanced glycation end-product-specific receptor; HGNC: 320). It was later discovered that RAGE also binds to multiple other soluble ligands, including S100 proteins, HMGB1, β-amyloid fibrils, complement protein C1q, and DNA/RNA, as well as associates with the transmembrane proteins TLR4 and β2-integrin Mac-1 [3,4,5,6,7,8,9]. Because of its broad binding capabilities, RAGE is regarded as a multi-ligand receptor. Studies have demonstrated that RAGE activation by its ligands leads to increased activation of the pro-inflammatory NFκB transcription factor pathway. The promoter region of AGER and several RAGE ligands contain NFκB regulatory elements, resulting in the upregulation of RAGE, its ligands, and pro-inflammatory cytokines [10,11,12,13]. This leads to a self-sustaining feed-forward loop, in which the initial activation of RAGE results in sustained activation of RAGE signaling, thereby generating an inflammatory environment. This has been shown to have detrimental effects in disease states such as diabetic complications, several cancers, Alzheimer’s disease, and vascular diseases [14,15,16,17,18].

Full-length RAGE consists of one V(variable)-type and two C(constant)-type immunoglobulin (Ig)-type folding domains (V-C1-C2) forming its extracellular domains, a single pass transmembrane domain (TM), and a short cytoplasmic tail (Cyto) [19,20,21,22]. The V- and C1-domains form a structural unit, while the C2-domain is connected by more flexible linkers to the C1 domain and the transmembrane domain [23]. A single membrane-spanning helical domain connects to the intracellular cytoplasmic tail. This domain is characterized by its short length of only 42 amino acids, a high content of positively charged amino acid arginine residues in the membrane-proximal region, followed by a glutamic acid-rich sequence. The cytoplasmic tail is required for transducing extracellular ligand binding into intracellular signaling. [22,24,25,26].

Studies have also shown that RAGE-expressing cells had increased adherence and spreading in the presence of extracellular matrix (ECM) proteins [27,28]. Amino acid sequence comparison has identified activated leukocyte cell adhesion molecule (ALCAM) as its closest homolog, with ~30% sequence homology. Based on structure comparisons, basal cell adhesion molecule (BCAM) has been identified as phylogenetically most closely related to RAGE [29]. ALCAM and BCAM mediate both heterotypic and homotypic cell–cell interactions, as well as interactions with extracellular matrix proteins. Because it had previously been observed that RAGE overexpression alters the cell adhesion properties, it was postulated that RAGE itself may have cell adhesion properties similar to those of ALCAM or BCAM [27,29].

However, the differences in ectodomain organization between RAGE (VCC-Ig-type domains) and ALCAM or BCAM (both with VVCCC-Ig-type domains) are significant. We therefore aimed to investigate the cell adhesion properties of RAGE in more detail and to assign functional roles to each of its Ig domains. Specifically, we intended to clarify whether the ectodomain of RAGE functions directly as a cell adhesion molecule or whether downstream events are critical. To investigate this knowledge gap, we used a protein engineering approach in which individual domains were deleted, and the cellular adhesion characteristics were studied. After finding that the cytoplasmic domain of RAGE plays a crucial role in modulating cell adhesion, we employed a proteomics approach and identified ITGA8 and several other adhesion-related proteins as being regulated by RAGE.

## 2. Materials and Methods

### 2.1. Plasmid Construction

Cloning of RAGE deletion constructs was achieved using NEBuilder HiFi DNA Assembly methodology (New England Biolabs, Ipswich, MA, USA) and cloned into the pcDNA3 vector. A previously generated pcDNA3_Fl_RAGE plasmid was used as a PCR template. Plasmids were amplified in E. coli DH5α, and sequences were confirmed through Edman DNA sequencing. All constructs retained the native RAGE signal peptide to facilitate membrane localization. Details of the PCR primers used, as well as the sequence of the final constructs and their corresponding amino acid sequences, are summarized in Supplementary Materials Table S1. Plasmids were purified using a plasmid miniprep kit (Omega, #D694, Norcross, GA, USA). The homogeneity and integrity of the isolated plasmids were confirmed by UV-VIS spectrometry and agarose gel electrophoresis, respectively. The plasmid stocks were prepared in batches and stored at −80 °C.

#### 2.1.1. Cell Culture

Human embryonic kidney (HEK293; ATCC, CRL-1573) and Chinese hamster ovary (CHO-K1; ATCC, CCL-61) cells were purchased from ATCC (Manassas, MA, USA) and cultured in Dulbecco’s Modified Eagle Medium (DMEM) complete media (ATCC, 30-2002™, Manassas, VA, USA) containing 10% Fetal Bovine Serum (FBS) (ATCC, 30-2020™) and 1% penicillin/streptomycin (Corning, Cat#30-002-CI, Corning, NY, USA). Cells were grown in a humidified incubator at 37 °C in an atmosphere containing 5% CO_2_. HEK293 cells stably expressing full-length RAGE (RAGE HEK293) were a generous gift from Dr. Heizmann (University of Zürich, Zürich, Switzerland) and were cultured in DMEM complete media with 0.5% vol/vol of G418 sulfate (Corning, Cat# 30-234-CI). MiaPaca2 (ATCC, CRM-CRL-1420) cells were cultured in DMEM complete media containing 2.5% horse serum (ATCC, 30-2040). Cells were routinely tested for mycoplasma using a two-stage PCR method as described in [30].

#### 2.1.2. Transient Transfection

Lipofectamine 3000 (Invitrogen, Carlsbad, CA, USA) was used for transfections. In brief, cells at a density of 5 × 10^5^ per 2 mL were seeded in a 6-well plate and allowed to grow until they reached 80–90% confluency. Before performing transfection, the media in the wells was replaced with fresh DMEM media. For each well, 3.0 μg of DNA diluted in 250 μL of Opti-MEM reduced serum media (Gibco, Waltham, MA, USA) was used to transfect the cells. The DNA-lipofectamine mixture in the wells was left for 8 h and then replaced with complete DMEM media.

#### 2.1.3. SDS-PAGE and Western Blotting

Cell lysates for SDS-polyacrylamide gel electrophoresis were prepared as follows: Transfected cells were harvested by scraping into ice-cold Phosphate-Buffered Saline (PBS), then centrifuged at 200 g at 4 °C. The pellet was resuspended in RIPA lysis buffer (1% (*v*/*v*) NP-40, 12 mM sodium deoxycholate, 0.1% SDS, and 1 mM protease inhibitor cocktail Calbiochem Set IV) and incubated on ice for 45 min, followed by centrifugation at 13,400 g at 4 °C. Protein concentrations in supernatants were determined using the Pierce BCA protein assay kit (Thermo Fisher, Waltham, MA, USA). In general, 10–100 µg of total protein was resolved on a 10, 12, or 15% SDS PAGE gel and transferred onto a 0.2 µm nitrocellulose membrane (Bio-Rad Cat# 1620112,Hercules, CA, USA) by wet-electroblotting for 1 h at 160 mA. The blot was blocked using 5% fat-free milk in Tris-buffered saline TBS containing 0.1% Tween 20 (TBS-T) for one hour at room temperature. Incubation with antibodies was performed at the recommended dilutions overnight in a cold room (4 °C) with gentle rocking. Blots were washed with TBS containing 0.1% Tween and incubated with HRP-conjugated secondary antibodies (Jackson ImmunoResearch, West Grove, PA, USA) raised against the primary antibodies at a 1:50,000 ratio for 1 hr at room temperature. An ECL luminescence substrate (Bio-Rad ECL Western Blotting substrate, Cat# 170-5061, Hercules, CA, USA) was used for visualizing the protein on the membrane. Antibodies against actin (Santa Cruz Biotechnology sc-1616, Dallas, TX, USA, and Cell Signaling Technologies #4970, Danvers, MA, USA) were used on the same membrane after stripping and served as the loading control.

#### 2.1.4. Immunofluorescence

Immunofluorescence images were obtained using either 35 mm m-Dishes (IBIDI, Fitchburg, WI, USA) or 18 mm × 18 mm cover glasses (Zeiss, Oberkochen, Germany) coated with collagen I (50 μg/mL) in 0.02 M acetic acid and incubated for 2 h at room temperature. Cells were then seeded onto the coated surfaces and allowed to reach 70% confluency. After 24 hours of transfection, cells were fixed for 10 min using 4% paraformaldehyde at RT and washed twice in ice-cold PBS. Permeabilization was performed with 0.1% Triton X-100 for 15 min on ice. Blocking was performed using 5% bovine serum albumin (BSA) containing 0.1% Tween-20 in PBS for 30 min at room temperature. Incubations with primary antibodies were performed at recommended dilutions in blocking buffer overnight in the cold room. The cells were washed three times with PBS containing 0.1% Tween for 5 min, followed by incubation with FITC-conjugated secondary antibodies (Jackson ImmunoResearch) diluted in blocking buffer (1:100) for 1 h at room temperature. The Hoechst 33342 stain (Invitrogen, Waltham, MA, USA) was used to stain the nucleus. Images were captured using a Zeiss confocal microscope (LSM700) and an Olympus FV3000 confocal laser scanning microscope.

#### 2.1.5. Flow Cytometry

Cells were detached using Cell Stripper (Corning, cat# 25-056-CI) solution and were washed three times in ice-cold PBS. After resuspending the cells in ice-cold PBS containing 2% FBS, the cells were counted, and 1 × 10^6^ cells were used for each sample. Cells were maintained at 4˚C throughout the labeling procedure. In brief, cells were fixed in 4% paraformaldehyde; subsequently, the fixed cells were either permeabilized with 0.1% Triton X-100 or left non-permeabilized. Blocking with 5% FBS in PBS for 30 min was more efficient than using 5% BSA. As the final step, cells were incubated with the primary antibody at the manufacturer’s recommended dilution, followed by labeling with a fluorophore-conjugated secondary antibody (Jackson ImmunoResearch, cat#115-095-062, or Cell Signaling Technologies, cat#4412) at 1:200 in blocking buffer. The non-transfected and the mock-transfected cells were treated equally and used as controls for the flow cytometry experiments. All experiments were performed using a BD Accuri C6 plus system (BD Biosciences, Franklin Lakes, NJ, USA). The default threshold was set to FSC-H (10,000) to exclude debris. The threshold was set in the Accuri software, and the program was configured to limit counts to 25,000 per sample. The data were analyzed using a gating strategy that excluded singlet cells, and the percentage of fluorescent cells in the analyzed sample was determined using a fluorescence intensity cut-off derived from mock and non-transfected cells. Events were displayed as FL1-A versus FSC-A to assess fluorescence intensity relative to cell size and to distinguish transfected (fluorescent) cells from non-transfected populations.

Details of the primary antibodies used in this study, along with dilution specifications for Western blotting (WB), immunofluorescence (IF), and flow-cytometry (FC) experiments, are mentioned in the Supplementary Materials Table S2.

### 2.2. Cell Adhesion Assay

#### 2.2.1. Using the Traditional Plate Assay

The assay was performed as described in [27,29] with some modifications. Tissue culture plates were coated with different extracellular matrix (ECM) proteins, R&D Systems, Minneapolis, MI, USA; Matrigel (10 µg/mL; R&D Systems #343300101), collagen IV (5 µg/mL; R&D Systems #341001001), collagen I (50 µg/mL; R&D Systems #344702001), and fibronectin (5 µg/mL; R&D Systems #342000101) for 2 hours at room temperature. The ECM-coated wells were washed with 5% BSA to reduce non-specific binding, and 50 × 10^3^ cells transfected with RAGE variants or mock-transfected cells were seeded in 100 μL of DMEM complete media. For the traditional plate assay, the cells were incubated for 5, 10, and 20 min, respectively, and then carefully washed twice with ice-cold PBS. To the washed wells, 100 μL of fresh DMEM complete media and 10% *v*/*v* of a 1 mg/mL resazurin solution were added. The plate was then incubated for 3 h. The resazurin fluorescence was measured at 560/590 nm (excitation/emission) using the SpectraMax multi-plate reader, and the percentage of adhered cells was calculated using the formula below:

% cells adhered = (Fluorescence from (washed well)/(non-washed well)) × 100

#### 2.2.2. Using the Xcelligence Real Time Cell Analyzer (RTCA)

The Xcelligence RTCA apparatus was set up as described in [31]. Plastic E16 well plates were coated with ECM proteins as described above, and the wells were washed with 5% BSA. The wells were seeded with 50 × 10^3^ cells transfected with RAGE variants or mock-transfected cells in 100 μL of DMEM complete media, and cell adhesion was monitored for 6 h. The cell impedance (Z) signals obtained from all RAGE variant-expressing cells using the Xcelligence real-time DP system software were compared with those from mock-transfected cells. The increase in the cell impedance value directly corresponds to increased cell adhesion in the corresponding wells. For each experiment, three well-replicated samples were used.

#### 2.2.3. Cell Spreading Assay

A total of 12 well chambered microscopy slides (Ibidi, cat# 81201, Bavaria, Germany) were coated with ECM proteins, as indicated in the cell–matrix adhesion assay. The cells, at a density of 3 × 10^4^, were seeded in 250 μL of media. After 3 h of incubation at 37 °C, the slide was imaged with a Leica DM2000 microscope at 20× magnification. ImageJ (version 1.54p; National Institutes of Health, Bethesda, MD, USA) was used to analyze the acquired images. The original image was converted to a binary image, and the shape of individual cells was traced using the free-hand tool. The spread area and perimeter for each cell were obtained using ImageJ [32]. The circularity of the cells was determined by using the formula below.

Circularity = 4π × (area/(perimeter^2^))

A circularity value of 1 indicates a perfect circular shape of the cells. The BSA-coated surface was used as the control. Per experiment, three independent wells were used as biological replicates. For each well, three representative images were acquired under identical imaging conditions, and all images were included in the quantitative analysis.

#### 2.2.4. RNA Extraction and cDNA Synthesis

Total cell RNA was extracted using the PureLink RNA Mini Kit (Invitrogen, Thermo Fisher Scientific, cat# 12183020, Waltham, Massachusetts, USA) according to the manufacturer’s protocol. The concentration of the RNA was measured by UV absorbance at 260 nm, and the ratio of absorbance at 260 nm to 280 nm (A260/A280) was calculated to estimate protein carryover into the RNA preparation. Extracted RNAs with a 260/280 nm ratio ≥ 2 were used for further analysis. The integrity of the extracted RNA was further confirmed using agarose gel electrophoresis (refer to Figure S1 from the Supplementary Materials). A total of 1 µg of RNA was used for immediate reverse transcription using Moloney Murine Leukemia Virus (M-MulV) Reverse Transcriptase (NEB cat# M0253, Ipswich, MA, USA) and an oligo(dT) primer.

#### 2.2.5. Quantitative Real-Time PCR (qRT-PCR)

A total of 20 ng of cDNA and 250 nM of gene specific forward and reverse primers were used in each PCR reaction (20 µL). HOT FIREPol EvaGreen qPCR master mix (ROX) (Solid BioDyne cat# 08-24-00001, Tartu, Estonia) was used. The total volume of the PCR reaction was 20 μL. The PCR thermal cycle program consisted of an initial denaturation step at 95 °C for 0:30 min, followed by 40 cycles of denaturation at 95 °C for 0:30 min, then annealing at 58 °C for 0:30 min, and extension at 72 °C for 1:00 min. The reactions were performed on a Stratagene Mx3000P Multiplex Quantitative PCR (QPCR) System, and cycle threshold (Ct) values were determined using the instrument’s software.

To calculate the fold change in the gene of interest (GOI), a double delta Ct (∆∆Ct) method was applied [33]. Details of all gene-specific primers used in this study are provided in the Supplementary Materials Table S3.

### 2.3. Cell Surface Proteomics

#### 2.3.1. Cell Surface Glycoprotein Biotinylation and Enrichment

Cell surface protein capture from live cells was performed as described in [34,35] with some modifications. Wild-Type HEK293 cells (WT HEK293) and RAGE expressing HEK293 cells (RAGE HEK293) were dissociated using CellStripper (Corning Cat# 25-056-CI) and washed twice with ice cold PBS by centrifuging at 100× *g* for 2 min at 4 °C. Cells (1.5 × 10^7^) were initially subjected to a mild oxidation reaction using 1.25 mM sodium metaperiodate (Biosciences) in PBS (pH 6.7) for 30 min. During this reaction, the vicinal hydroxy group in carbohydrates undergoes oxidative ring-opening, forming a reactive aldehyde. The reaction was quenched using 1 mM ethylene glycol for 30 min, followed by washing the cells three times with ice-cold PBS. The biotin labeling reaction was carried out at 4 °C using 250 µM aminooxy biotin (Biotium 90113, Fremont, CA, USA) and 10 mM aniline (Sigma, Burlington, MA, USA) as a catalyst for 90 min [35].

The cells were then washed four times with ice cold PBS and four times with PBS containing 1 mM EDTA. Cells were lysed in lysis buffer (10 mM Tris, 150 mM NaCl, 1% Triton X-100, and 1 mM Calbiochem cocktail set VII protease inhibitor) followed by five cycles of five bursts of sonication and incubation in the cold room with gentle rocking for 3 hrs. The lysates were centrifuged at 20,000× *g* for 20 min at 4 °C. This step was repeated twice by adding half the volume of the lysis buffer used in the first lysis step to the pellet to extract remaining proteins from the debris. The supernatant from the lysate was collected, centrifuged again to remove residual debris, and immediately processed for cell surface protein isolation and enrichment using NeutrAvidin Gel (Prod# 1859388, Pierce, Waltham, MA, USA). The beads were equilibrated with PBS, and 3 mg of total biotinylated protein was added. The capture reaction was then incubated overnight with gentle rocking at 4 °C. The next day, the beads were washed using a series of buffers with protease inhibitors at 4 °C. The first four washes were performed with lysis buffer, followed by PBS containing 0.1% Tween and 50 mM ammonium bicarbonate. In total, 16 washes were performed, with the last two washes using 50 mM ammonium bicarbonate without protease inhibitors.

#### 2.3.2. Sample Processing for Mass Spectrometry (MS)

To reduce disulfide bonds to free cysteine, the washed beads were reduced using 20 mM DTT in 50 mM ammonium bicarbonate containing 0.1% RapiGest SF (Cat# 186001860, Waters, Milford, MA, USA) for 1 h at 37 °C. The beads were then alkylated with 50 mM iodoacetamide for 1 h at room temperature, followed by quenching the reaction with 20 mM Dithiothreitol (DTT) for 15 min. Excess reagents were washed out, and on-bead protein digestion was performed using a 1:50 (*w*/*w*) ratio of protein to trypsin (Pierce Trypsin Protease, MS Grade 90057, Waltham, MA, USA) for 24 h at 37 °C. The supernatant from the on-bead digestion reaction was collected, and RapiGest was precipitated by adding 0.5% (*v*/*v*) trifluoroacetic acid. After centrifugation, the supernatant was collected, lyophilized, and stored at −80 °C. A brief C18 column (100 µL bed) purification of the digestion peptides was performed using Pierce™ C18 Tips (cat# 87784) following the manufacturer’s instructions.

#### 2.3.3. Nano LC-MS Acquisition

A Waters Acquity nanoUPLC system, equipped with an autosampler (Waters, Milford, MA, USA), was used for the chromatographic separation of the digested peptides. Peptides were loaded on a trapping column (Waters Acquity UPLC M-class Symmetry, C18, 5 µm, 180 µm × 20 mm) at a flow rate of 15 µL/min for 3 min with 99.5% solvent A (0.5% (*v*/*v*) formic acid in water) and 0.5% solvent B (0.5% (*v*/*v*) formic acid in acetonitrile). After trapping, peptides were resolved on a Waters analytical column (Acquity UPLC M-class HSS T3, 1.8 µm, 100 µm × 100 mm, Milford, MA, USA) at a flow rate of 0.4 µL/min using a linear gradient of solvents A and B. Initial %B increased from 2% to 5% over 1 min, held at 5% for 4 min, then increased to 25% over 120 min, to 32% over 20 min, and further to 95% over 1 min. After holding at 95% for 5 min, %B was returned to 2% over 1 min and maintained at this value for 18 min to facilitate re-equilibration.

For mass spectrometry, a hybrid quadrupole-Orbitrap Q Exactive mass spectrometer I (Thermo Fisher Scientific) was used. The LC eluent was ionized using a nanoelectrospray ion source in positive ionization mode, with an electrospray voltage of 1.8 kV. MS/MS data were collected in data-dependent acquisition mode with dynamic exclusion (30 s). Profile full MS scans were collected at 70,000 resolution (*m*/*z* 400–1600) and a maximum of 10 centroid product ion scans at 17,500 resolution per cycle. Fragmentation was performed by collision-induced dissociation (CID) at 30% collision energy. AGC targets were 1 × 10^6^ ions for full scans and 1 × 10^5^ for MS/MS scans.

#### 2.3.4. MS-Data Processing

For protein identification, Protein Discoverer (version 2.2, Thermo Fisher Scientific) was used to search MS/MS spectra and parent proteins against the Homo Sapiens reference proteome database (UniProtKB/Swiss-Prot; Proteome ID UP000005640), downloaded on 10 September 2021. Minora Feature Detector was used for unlabeled quantification. Initial mass deviation of precursor ion and fragment ions was up to 10 ppm and 0.5 Da, respectively. Enzyme specificity was set to trypsin with a maximum of two miscleavages allowed. Minimum peptide length was set to 6, and maximum to 150 amino acids. Carbamidomethyl-Cys was set as a static modification. Met oxidation, protein *N*-terminal acetylation, and biotin-Lys were set as dynamic modifications. The expression of identified surface proteins was normalized using Na, K-ATPase alpha 1 subunit (ATP1A1) (UniProt entry: P05023), a prominent cell surface protein marker [36,37] that had a similar calculated abundance of protein expression in both WT HEK293 and RAGE HEK293. The fold change was represented by plotting the changes in the expression ratio between the two samples, with the WT HEK293 group serving as the calibrator and its expression level set to 1.

#### 2.3.5. Statistical Analysis

Data are presented as mean ± standard deviation (SD) of biological replicates. For in vitro cell culture assays, each biological replicate was performed with 3 to 6 technical replicates. Statistical analyses were performed using a two-tailed Student’s *t*-test (for comparisons between two groups) or one-way ANOVA with a Tukey post hoc test (for comparisons among multiple groups). For the RTCA (real-time cell adhesion) experiments, a two-way ANOVA (Variant × Time) followed by Sidak’s post hoc was employed to include time as a categorical independent variable. *p*-values of less than 0.05 were considered statistically significant, with significance levels indicated as follows: * *p* < 0.05; ** *p* < 0.01; *** *p* < 0.001.

## 3. Results

### 3.1. Expression and Localization of RAGE Domain Deletion Variants

**Figure 1:**
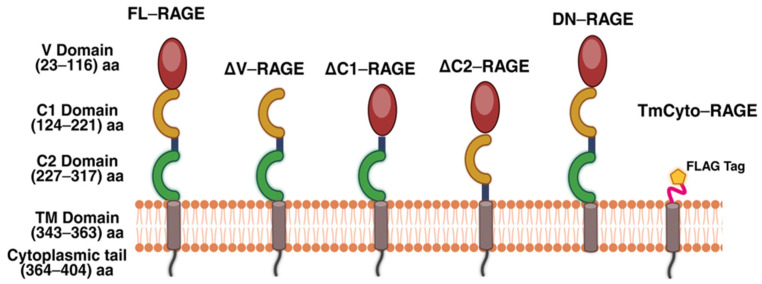
Domain composition of full-length RAGE (FL-RAGE) and the domain deletion RAGE variants used in this study. FL-RAGE consists of the *N*-terminal V domain followed by two C-domains (C1, C2), a transmembrane region (TM), and a cytoplasmic tail (CT). The other RAGE variants lack the indicated RAGE domain(s). Created with BioRender.com.

Plasmids encoding full-length (FL)–RAGE and the engineered RAGE deletion variants shown in Figure 1 were generated and sequence confirmed. The ΔV (deleted V)–domain variant lacks residues 23–101; the ΔC1 (deleted C1)–domain variant lacks residues 117–221; the ΔC2 (deleted C2)–domain variant lacks residues 221–318; the dominant negative (DN–) variant lacks residues from 364 to 404; the TmCyto– variant lacks residues 23–318.

**Figure 2:**
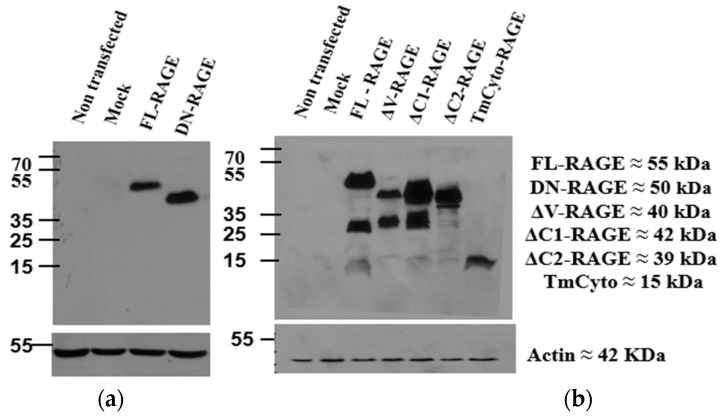
Protein expression of FL-RAGE and other RAGE variants in HEK293 cells. (**a**) The blot on the left used anti–RAGE 9A11, an antibody recognizing the V-domain of RAGE. (**b**) The blot on the right used RAGE D1A12, an antibody recognizing the C-terminal domain of RAGE.

The expression of the above RAGE constructs in HEK293 cells was analyzed by Western blots using anti-RAGE antibodies (Figure 2). HEK293 cells do not express endogenous RAGE, and none was detected on blots of non-transfected cells or cells transfected with pcDNA3 without a RAGE insert (mock transfected cells). Transfection with FL–RAGE resulted in a single clear band of approximately 55 kDa. This molecular weight is approximately 10 kDa higher than theoretically expected, but is typical for RAGE due to post-translational modifications and glycosylation [22]. The domain deletion variants of RAGE exhibited lower apparent molecular weights, consistent with their expected molecular weights.

Two different antibodies were used for Western analysis. The anti–RAGE 9A11 antibody (Santa Cruz Biotechnology) recognizes the *N*-terminal V–domain of RAGE and was used to detect the RAGE deletion variant, which lacks the cytoplasmic domain (DN–RAGE). The other RAGE variants were analyzed using anti–RAGE D1A12 antibody (Cell Signaling Technology, Danvers, MA, USA), which recognizes the cytoplasmic domain of RAGE. Using this antibody, there was an indication of minor proteolysis in the deletion variants in which either the V– or the C1–domain was deleted. There was also some indication of proteolytic cleavage of full-length RAGE, and it was observed to increase with time (refer to Supplementary Materials Figure S2). The molecular weight of the proteolytic fragment suggests cleavage between the C2–domain and the transmembrane region. It is well known that RAGE undergoes ectodomain shedding [24,25,38].

**Figure 3:**
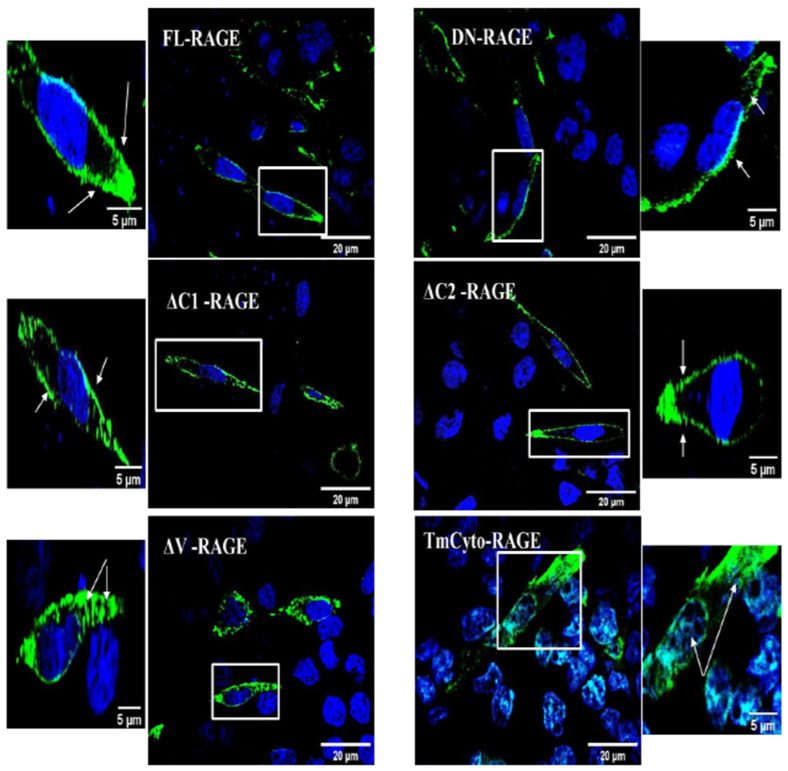
Confocal microscopy images of HEK293 cells expressing the FL-RAGE and different domain deletion variants. For the DN-RAGE construct, the anti-RAGE 9A11 antibody was used, while for all other variants, the RAGE D1A12 antibody was used. Enlarged images with arrows indicate surface and intracellular localization in the RAGE variants. Additional images are included in the Supplementary Material Figure S3.

We next employed immunofluorescence imaging to investigate the cellular localization of FL–RAGE and its domain deletion variants (Figure 3). FL–RAGE, DN–RAGE, ΔC1–, and ΔC2– localize exclusively to the plasma membrane. The ΔV– and TmCyto–variants had plasma membrane and some intracellular localization. RAGE has previously been shown to have some intracellular localization in melanoma cells and T-cells [39,40].

**Figure 4:**
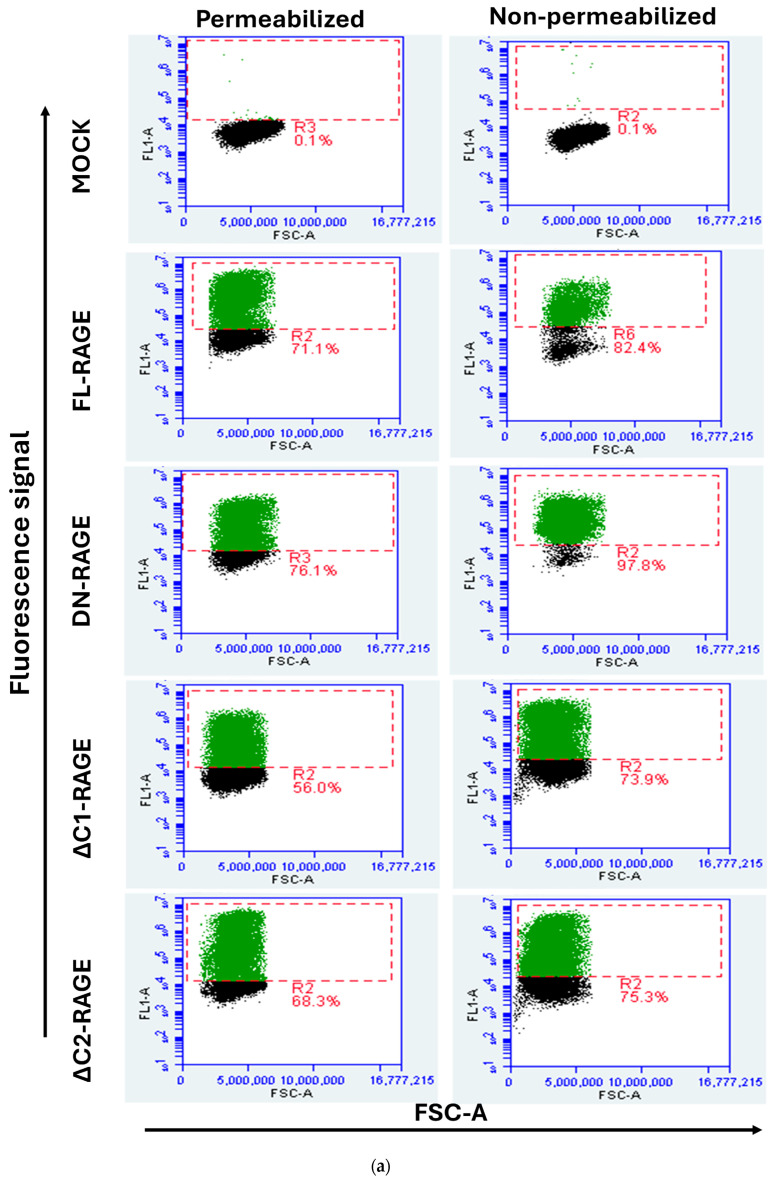

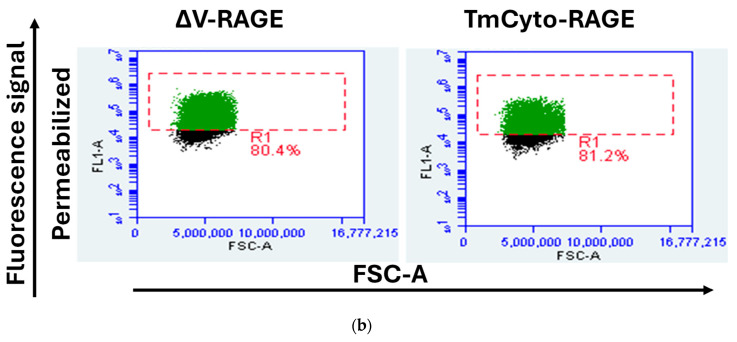
Representative flow cytometry dot plot displaying FSC-A (x-axis) versus fluorescence signal (FL1-A) (y-axis) of HEK293 cells transfected with RAGE variants. The gated cell population in green color indicates cells transfected with RAGE variants. (**a**) The left column shows flow cytometry dot plots for the non-permeabilized condition using RAGE 9A11 antibody recognizing its N-terminal, and the right column shows those for the permeabilized condition using RAGE D1A12 recognizing its C-terminal (*n* = 3); (**b**) The dot plot in the bottom represents HEK293 cells transiently transfected with ΔV-RAGE and TmCyto-RAGE under permeabilized conditions using anti-RAGE D1A12 antibody (*n* = 2). One representative example of the experiments performed is shown.

The relative cell surface localization of FL-RAGE and its domain deletion variants was quantified using flow cytometry. Cells were analyzed either with their plasma membrane intact to quantify cell-surface-localized RAGE or after membrane permeabilization, which allows antibody binding to both intracellular and cell-surface-exposed RAGE. The results are presented in Figure 4 and Table 1. The data show that FL-RAGE and ΔC2-RAGE are almost exclusively expressed on the cell surface, 85% and 80% of the cell surface expression, respectively. DN-RAGE and ΔC1-RAGE showed similarly high levels of surface localization 78% and 75%, respectively. Analysis of the ΔV-RAGE variant was not attempted because we were unable to identify an antibody that would bind the RAGE C1- or C2-domain. The TmCyto-RAGE construct lacked a detectable cell surface domain. However, we were able to determine the percentage increase in FL1-A signal in ΔV- and TmCyto-RAGE compared to mock using the anti-RAGE D1A12 antibody in permeabilized conditions. The signal was similar to the signal from FL-RAGE under the same conditions.

### 3.2. The Presence of Only the Cytoplasmic Domain of RAGE Enhances Cell Adhesion to Extracellular Matrix (ECM) Proteins

**Figure 5:**
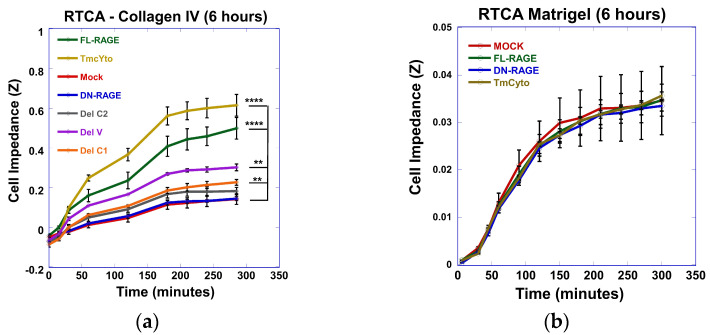
Plot of Xcelligence RTCA data showing cell adhesion of HEK293 cells transiently transfected with RAGE variants to collagen IV (**a**) and to Matrigel-coated surfaces (**b**) over 6 h. The graph represents the average values from three independent experiments (*n* = 3), with data shown as mean ± SD. Statistical analysis was performed using two-way ANOVA with a Sidak’s post hoc; *n* = 3 experimental replicates; ** *p* < 0.01, **** *p* < 0.0001.

Data from the RTCA on the collagen IV-coated surface (Figure 5a) showed that FL-RAGE-transfected HEK293 cells exhibited a steady increase in cell impedance over time. The effect of time on cell adherence was considered during statistical analysis of the data and was found to be highly significant. The time dependence of initial cell adherence is well known and is not further analyzed in this work. At the 6 hours (300-minute) time point, the FL-RAGE-expressing cells reached an impedance (Z) of 0.5 ± 0.05 Ω, which was significantly higher than in the mock (**** *p* < 0.0001), and a significant difference between the domain deletion variant was apparent. Deleting individual domains in the extracellular region of RAGE reduced cell impedance compared with the FL-RAGE variant. In particular, deleting the C1 or C2 domain (ΔC1 and ΔC2) of RAGE resulted in lower impedance values of 0.13 ± 0.02 and 0.15 ± 0.015 Ω, respectively, compared to deleting the V domain (ΔV), which had a slightly higher impedance, Z = 0.2 ± 0.017 Ω at 6 h.

Surprisingly, the removal of the cytoplasmic domain, in the case of the DN-RAGE construct, appeared to affect cell adhesion. The cell impedance over time followed a pattern identical to that of the mock-transfected cells (0.1 ± 0.02). This suggests that the extracellular domains of RAGE play a minimal role in modulating cell adhesion to collagen IV. The above observation was further supported by the fact that the TmCyto variant, which lacks the entire ectodomain of RAGE, exhibited adhesion behavior comparable to that of FL-RAGE. The impedance value from TmCyto HEK293 cells (0.6 ± 0.05) exceeded that of the FL-RAGE HEK293 cells after 6 h. The change in impedance in TmCyto-RAGE was found to be statistically significantly different compared to that of the mock (*p* < 0.0001). This suggests that the extracellular domains modulate cell adhesion mediated by RAGE; they are not strictly required, as the cytoplasmic domain alone can mediate adhesion.

Interestingly, repeating the adhesion experiment in an RTCA with a Matrigel-coated surface showed no significant change in impedance over time with (FL-, DN-, TmCyto-) RAGE variants and the mock-transfected cells (Figure 5b). It was also noted that the Z value after 6 h in FL- and TmCyto-RAGE variants was 10 times lower on a Matrigel-coated surface (Z = 0.03 Ω) compared to collagen IV. Additionally, the plots indicated that the impedance after the 3-hour time point did not exhibit a steady increase but instead showed a somewhat stationary adhesion phase.

**Figure 6:**
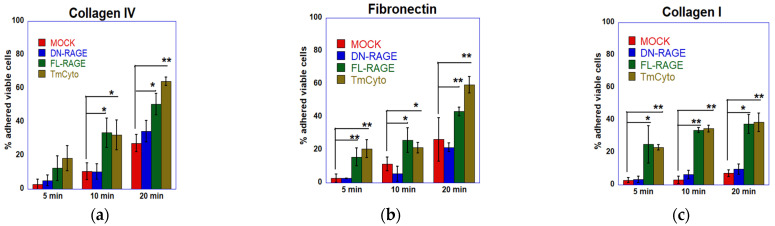
Cell adhesion of FL-RAGE, DN-RAGE, TmCyto-RAGE, and mock transfected WT HEK293 cells to different ECM proteins. The top row represents the plots from the traditional plate cell adhesion assay to (**a**) collagen IV, (**b**) fibronectin, and (**c**) collagen I. These plots represent the average of three independent experiments (*n* = 3), with data shown as mean ± SD. Statistical analysis was performed using one-way ANOVA with Tukey’s post hoc test; * *p* < 0.05, ** *p* < 0.01.

Further, a cell adhesion plate assay was performed to compare the adhesion trends to collagen IV from the RTCA DP system for (FL-, DN-, TmCyto-) RAGE variants and mock-transfected cells (Figure 6a). The data showed a similar change in adhesion pattern, with the FL- and TmCyto variants exhibiting the highest adherence (57% and 65%) at the 20 min time point. While the adherence of DN-RAGE-expressing cells increased over time, it was not statistically significantly different from that of mock-transfected cells. Evaluating the adhesion of the three RAGE variants (FL-, DN-, and TmCyto-) to fibronectin (Figure 6b) and collagen I (Figure 6c) showed an adherence pattern similar to collagen IV.

Overall, the results demonstrate that the cytoplasmic domain is critical in RAGE-mediated cell adhesion to ECM proteins. It also suggests that RAGE preferentially adheres to different ECM proteins in the following order: collagen IV > fibronectin > collagen I > matrigel.

### 3.3. Enhanced Cell Adhesion by the Cytoplasmic Domain Is Followed by Increased Cell Spreading to ECM Proteins

**Figure 7:**
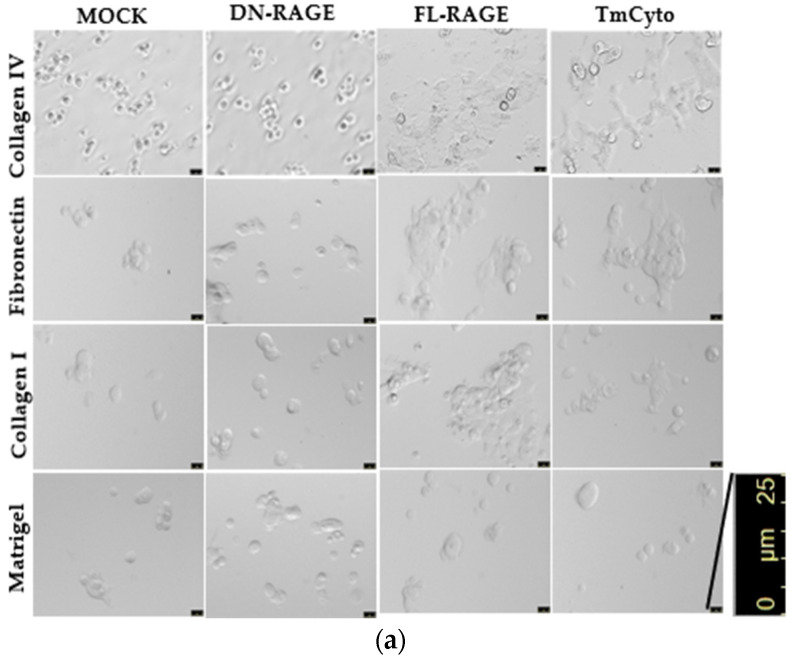

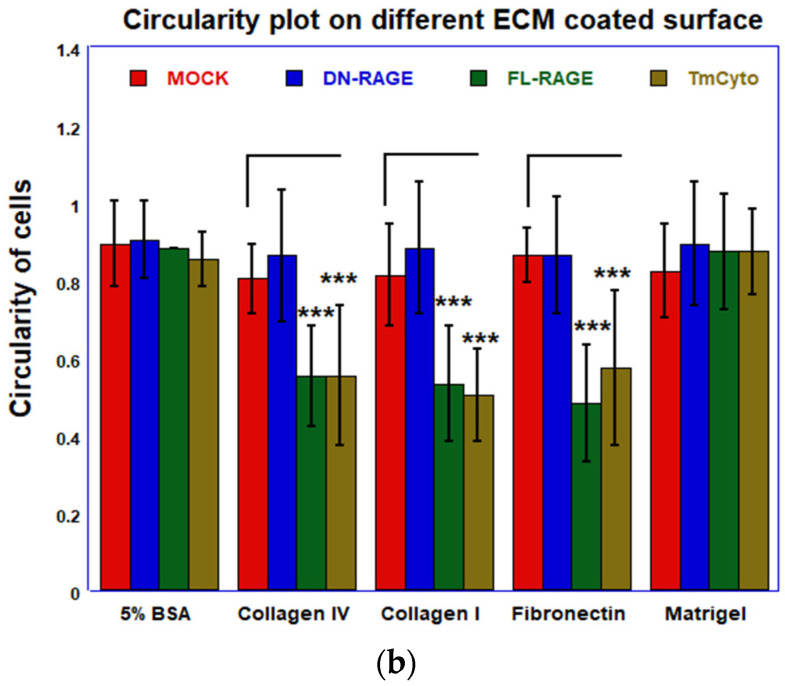
(**a**) Microscopy images from cell spreading assay of WT HEK293 cells transfected with mock and (FL-, DN-, and TmCyto-) RAGE variants. (**b**) Quantitative analysis of circularity of cells from cell spreading assay. The bar graph represents the average cell circularity values from two independent experiments (*n* = 2), with data shown as mean ± SD. For each condition, three replicate wells were analyzed, and three non-overlapping images were acquired per well (nine images total per condition). Statistical analysis was performed using one-way ANOVA with Tukey’s post hoc test; *** *p* < 0.001.

Images from the cell spreading assay revealed that FL-RAGE and TmCyto RAGE variants exhibited polarized cell morphology and covered the maximum surface area in collagen IV, fibronectin, and collagen I (Figure 7a). Although a few cells in the images exhibited a circular morphology, most cells had a dense, almost neuronal-like, spread pattern. The reason is likely due to transient transfection; not all cells express the protein at the same level. Mock- and DN-RAGE-transfected cells exhibited circular spread patterns, with most cells sparsely attached to the surface and covering a smaller area. Images from the matrigel-coated surface showed no change between the RAGE variants FL-, DN-, and TmCyto and the mock. Cells appeared circular in morphology and were observed to be spaced apart from one another.

Data from the cell spreading image analysis demonstrate that adhesion and spreading patterns are linked and occur in sequence. In the case of FL- and TmCyto-RAGE, enhanced cell adhesion is followed by increased cell spreading in all the ECM proteins used in the study, except for Matrigel. The adhesion data for the Matrigel-coated surface showed no significant RAGE effect, consistent with the spreading analysis on Matrigel. The images from the cell spreading assay were quantified by calculating cell circularity using ImageJ (Figure 7b). The circularity value for TmCyto- and FL-RAGE variants ranged between 0.5 and 0.55 for all the tested ECM proteins except for Matrigel (0.9). Mock and DN-RAGE, which appeared to be round in morphology, were observed with circularity values > 0.8. The BSA-coated surface served as the control, with the RAGE variants and mock showing similar circularity values of ~0.9.

### 3.4. qPCR Analysis Shows Selective Regulation of CAM Gene Expression in RAGE HEK293 Cells

The qPCR analysis of 68 genes mediating cell–cell and cell-extracellular matrix in WT HEK293 and RAGE HEK293 had a mean Cts between (21.0–33.0 ± SD). In these experiments, RAGE expression was used as an internal control. The average Ct value of RAGE WT HEK293 was 28.1 ± 1.8, compared to RAGE HEK293, which was 19.0 ± 1.4, corresponding to a 500-fold increase in RAGE expression in these cells. The CD36 gene had the highest Ct in both WT HEK293 (36.4 ± 1.7) and RAGE HEK293 (37.1 ± 1.5) cells, indicating lower transcriptional expression. The Ct values of other tested genes, belonging to different classes of cell adhesion molecules such as integrins, cluster of differentiation (CD) receptors, and the IgG receptor superfamily, showed no significant changes in expression between WT HEK293 and RAGE HEK293 cells (Supplementary Table S4). Relative gene expression levels were normalized to the housekeeping gene, phosphoglycerate kinase 1 (PGK1). The WT group served as the calibrator, and its expression level was set to 1. The fold change in the expression of the above tested cell adhesion genes between WT HEK293 and RAGE HEK293 varied by a factor of 1 ± 0.5.

**Figure 8:**
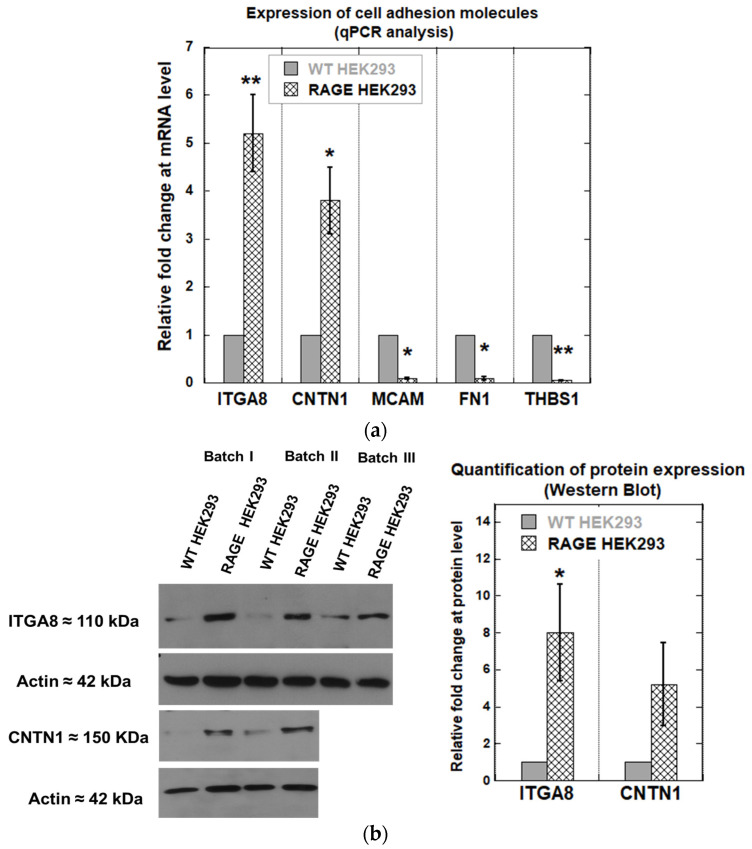
(**a**) Change in relative gene expression of cell adhesion–related genes (ITGA8, CNTN1, MCAM, FN1, and THBS1) between WT and RAGE HEK293 cells (*n* = 3). (**b**) Western blot against ITGA8 and CNTN1 using lysates from WT and RAGE HEK293. The bar plot shows quantification of ITAG8 (*n* = 3) and CNTN1 (*n* = 2) expression, with data expressed as mean ± SD. Statistical analysis was performed using a two-tailed Student’s *t*-test for ITGA8 only; * *p* < 0.05, ** *p* < 0.01.

Integrin alpha 8 (ITGA8), contactin 1 (CNTN1), melanoma cell adhesion molecule (MCAM), fibronectin 1 (FN1), and thrombospondin-1 (THBS1) were found to be differentially expressed between WT HEK293 and RAGE HEK293 (Figure 8a). ITGA8 and CNTN1 showed a statistically significant increase in fold change of 4.6 ± 0.8 and 3.1 ± 0.7 in RAGE-HEK293 samples. In contrast, MCAM, FN1, and THBS1 genes were found to be downregulated in RAGE HEK293 compared to WT HEK293. The changes in expression for these genes were as follows: MCAM, 0.089 ± 0.02; FN1, 0.45 ± 1.3; THBS1, 0.064 ± 1.1. Western blot analysis was performed for the ITAG8 and CNTN1 proteins to confirm whether the effect observed at the gene level also replicates at the protein level. It was observed that ITGA8 and CNTN1 showed increased expression on the protein level by about 8.0-fold for ITGA8 and about 5-fold for CNTN1, respectively (Figure 8b). The protein expression level of ITGA8 differed significantly between RAGE HEK293 and WT cells (* *p* < 0.05).

### 3.5. Cell Surface Proteomics on RAGE HEK293 Cells Shows Altered Expression of Adhesion-Relevant Proteins Compared to WT HEK293 Cells

Data analysis from three experiments revealed that nearly 50% of the adhesion-relevant proteins were detected in both WT and RAGE HEK293 cells. It was also observed that RAGE expression in HEK293 cells increased the expression of selective adhesion-relevant proteins that were absent in WT HEK293 cells. Comparing results within individual sample sets revealed that 29 proteins were identified across all three experiments in RAGE HEK293 samples and 24 in WT HEK293 samples (Supplemental Figure S4).

**Figure 9:**
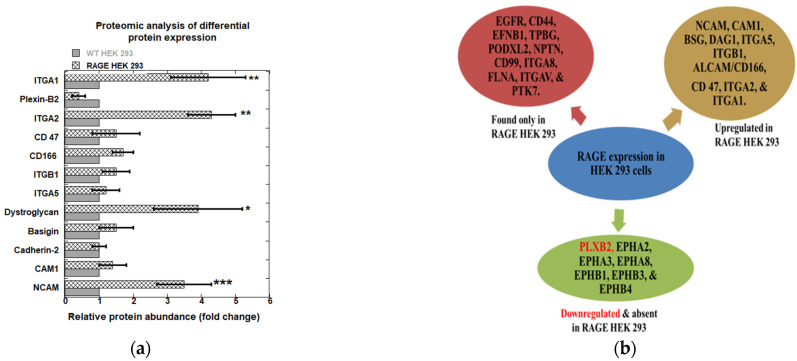
(**a**) Relative protein abundance (fold change) between WT HEK293 and RAGE HEK293 cells based on quantitative proteomics analysis. Protein expression levels were normalized to Na^+^/K-ATPase. Fold changes were calculated relative to WT HEK293 levels. Bars represent mean values from (*n* = 3) independent experiments, with data expressed as mean ± SD. Statistical analysis was performed using a two-tailed Student’s *t*-test; * *p* < 0.05, ** *p* < 0.01, *** *p* < 0.001. (**b**) Differential expression of cell surface proteins upon RAGE expression in WT HEK293 cells as identified by cell surface proteomics. Proteins were classified as upregulated, downregulated, uniquely present, or absent based on detection across samples.

From the total list of identified proteins of all three independent experiments 12 proteins were found in both WT and RAGE HEK293 which included neural cell adhesion molecule (NCAM), cell adhesion molecule 1 (CAM1), basigin (BSG), dystroglycan (DAG1), integrin alpha 5 (ITGA5), integrin beta 1 (ITGB1), activated leukocyte cell adhesion molecule (ALCAM/CD166), leukocyte surface antigen CD47 (CD 47), integrin alpha 2 (ITGA2), Plexin-B2 (PLXB2), cadherin-2 (CADH2), and integrin alpha 1 (ITGA1). Of these, cadherin-2 showed no change in the expression, and plexin-B2 was downregulated by half fold (0.5 ± 0.2) in RAGE HEK293. The remaining proteins were upregulated in RAGE HEK293 cells, and among these, dystroglycan, ITGA2, ITGA1, and NCAM showed fold changes greater than 3.5 ± 0.7 and were statistically significant (* *p* < 0.05, ** *p* < 0.01, *** *p* < 0.001). Adhesion proteins CAM1, basigin, ITGA5, ITGB1, CD166, and CD47 exhibited a slight increase in fold change, ranging from 1.5 to 2.0 (Figure 9a).

Interestingly, in RAGE HEK293 cells, a set of new cell adhesion proteins was identified that were not found in WT HEK293 cells: epidermal growth factor receptor (EGFR), CD44 antigen (CD44), ephrin-B1 (EFNB1), trophoblast glycoprotein (TPBG), podocalyxin-like protein 2 (PODXL2), neuroplastin (NPTN), CD99 antigen (CD99), integrin alpha-8 (ITGA8), filamin-A (FLNA), integrin alpha-V (ITGAV), and inactive tyrosine-protein kinase 7 (PTK7). Also, RAGE expression in HEK293 completely abolished the expression of a set of proteins that belong to ephrin type A and B receptors; ephrin type-A receptor 2 (EPHA2), ephrin type-A receptor 3 (EPHA3), ephrin type-A receptor 8 (EPHA8), ephrin type-B receptor 1 (EPHB1), ephrin type-B receptor 3 (EPHB3), and ephrin type-B receptor 4 (EPHB4) (Figure 9b). Using the Panther classification system, the functional classification of these differentially expressed genes for pathway analysis yielded the highest hits for integrin-mediated signaling pathways. Overall, our proteomics study suggests that RAGE expression in HEK293 cells differentially regulates the expression of adhesion-related surface proteins.

### 3.6. RAGE Knockdown Reduces Cell Adhesion and Cell Spreading to ECM by Downregulation of ITGA8 in RAGE HEK293 Cells and MiaPaCa2 Cells

**Figure 10:**
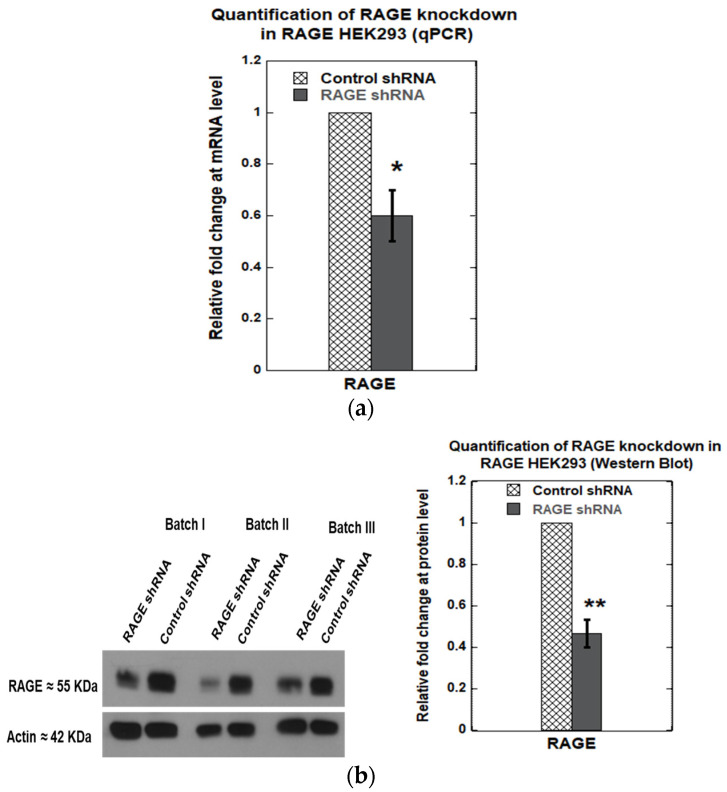
Relative fold change in RAGE expression after RAGE knockdown in RAGE HEK293 cells: (**a**) qPCR; (**b**) Western blot. The plots represent the average of three independent experiments (*n* = 3). Data are expressed as mean ± SD, and statistical significance was determined using a two-tailed Student’s *t*-test (* *p* < 0.05, ** *p* < 0.01). Anti-RAGE D1A12 antibody was used to detect RAGE in these samples.

**Figure 11:**
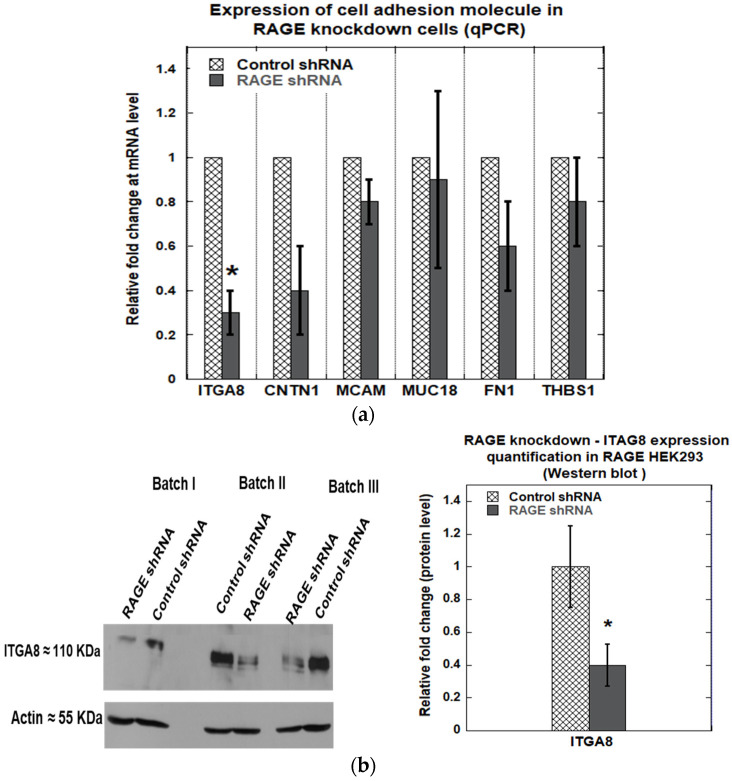
(**a**) qPCR analysis showing the relative expression of cell adhesion–related genes (ITGA8, CNTN1, MCAM, FN1, and THBS1) in control versus RAGE-knockdown in RAGE HEK293 cells; (**b**) Western blot against ITGA8 in control vs. RAGE knockdown samples. The bar plot shows the relative quantification of ITAG8 expression from three independent experiments (*n* = 3). Data are expressed as mean ± SD, and statistical significance was determined using a two-tailed Student’s *t*-test (* *p* < 0.05).

Knockdown of RAGE was performed using a shRNA plasmid targeting RAGE (Genecopoeia, product# HSH094823-CU6), and scrambled shRNA was used as a control (Genecopoeia, product# CSHCTR001-CU6). A knockdown efficiency of 40 ± 10% (Figure 10a) was observed at the transcript level and 54 ± 7% at the translational level (Figure 10b). Next, we investigated the effect of RAGE knockdown on cell adhesion molecules, including ITGA8, CNTN1, MCAM, FN1, and THBS1, which exhibited differential gene expression levels in RAGE HEK293 cells. We found that RAGE knockdown significantly downregulated ITGA8 expression by 70 ± 10% at the mRNA level (Figure 11a) and 60 ± 10% at the protein level (Figure 11b).

**Figure 12:**
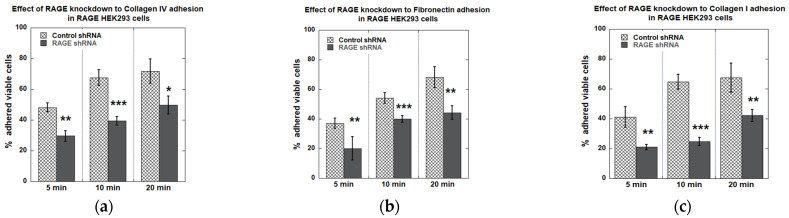
Plots from cell adhesion plate assay with RAGE HEK293 cells transfected with control/scrambled and RAGE shRNA to (**a**) collagen IV, (**b**) fibronectin, and (**c**) collagen I coated surfaces. This plot shows the average % of cells adhered across all three independent experiments (*n* = 3). Data are expressed as mean ± SD, and statistical significance was determined using a two-tailed Student’s *t*-test (* *p* < 0.05, ** *p* < 0.01, *** *p* < 0.001).

RAGE knockdown reduced the cell adhesion of RAGE HEK293 cells to different ECM proteins (Figure 12). For Collagen IV, collagen I, and fibronectin, RAGE knockdown reduced cell adhesion by 30–40% at the 20 min time point. At earlier time points, the reduction in cell adhesion was even more pronounced, except on fibronectin-coated surfaces (Figure 12).

**Figure 13:**
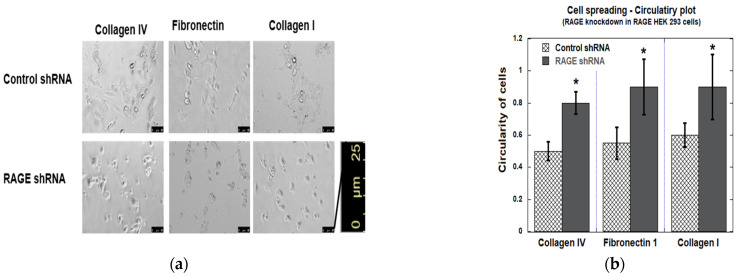
Cell spreading assay in RAGE HEK293 cells transfected with control and RAGE shRNA. (**a**) Results from the image analysis of cell spreading. (**b**) The bar graph represents the quantitative analysis of average cell circularity values from three independent experiments (*n* = 3), with data shown as mean ± SD. For each condition, three replicate wells were analyzed, and two non-overlapping images were acquired per well (six images total per condition). Statistical analysis was performed using a two-tailed Student’s *t*-test; *n* = three biological replicates and * *p* < 0.05. Scale bar 25 μm.

The data from cell spreading assays matched those of the adhesion assay (Figure 13). Overall, RAGE knockdown decreased the cell polarization, resulting in a more circular appearance, while control shRNA-transfected RAGE HEK293 cells maintained a dense, neuronal-like spread pattern typical of RAGE-expressing HEK293 cells. The observed circularity value for RAGE shRNA-transfected cells was >0.8, whereas the control shRNA-transfected cells had a value of <0.5.

**Figure 14:**
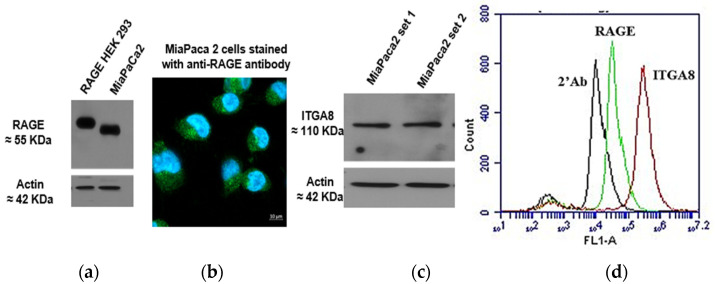
Validation of endogenous expression of RAGE and ITGA8 in MIA PaCa-2 cells. (**a**) Western blot against RAGE in RAGE HEK 293 and MIA PaCa-2 lysates; {(**b**) Immunofluorescence analysis of RAGE; (**c**) Western blot against ITGA8; (**d**) Histogram from flow cytometry analysis of cell surface RAGE and ITGA8 in non-permeabilized condition}-in MIA PaCa-2 cells.

To further investigate the functional dependency of RAGE and ITGA8, we used the pancreatic cancer cell line, MIA PaCa-2. Endogenous expression of RAGE and ITGA8 was determined in MIA PaCa-2 cells (Figure 14). Western blot analysis using the anti-RAGE D1A12 antibody revealed a RAGE band at approximately 55 kDa in the MIA PaCa-2 lysate. Immunofluorescence analysis showed that RAGE was predominantly localized in the intracellular region. ITGA8 expression was confirmed by Western blot and flow cytometry.

**Figure 15:**
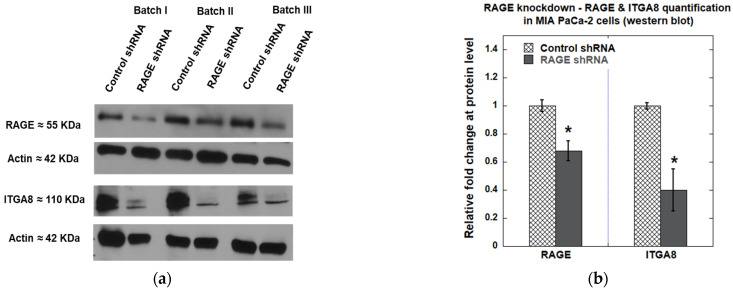
(**a**) Western blot showing expression of RAGE and ITGA8 in MIA PaCa-2 cells after RAGE knockdown; (**b**) The plot illustrates the quantification of the indicated proteins, shown as the average fold change from three independent experiments *(n* = 3). Data are expressed as mean ± SD, and statistical significance was assessed using a two-tailed Student’s *t*-test (* *p* < 0.05).

We observed approximately 30% knockdown of RAGE in MIA PaCa-2 cells using Western blot analysis. Furthermore, as observed in RAGE HEK293 cells, knocking down RAGE in MIA PaCa-2 cells also resulted in a 60% reduction in ITGA8 levels (Figure 15). Overall, our results showed that RAGE knockdown reduced cell adhesion and cell spreading, which might result from the downregulation of ITGA8.

## 4. Discussion

Based on our data from cell adhesion and cell spreading assays, RAGE was found to mediate cell adhesion only in part through its extracellular domain. Past studies on RAGE have suggested that its extracellular domains mediate cell–cell and cell–matrix adhesion. [10,29,41,42,43]. Our data demonstrated that the DN-RAGE variant, which contained all the extracellular domains of RAGE (V, C1, and C2), behaved similarly to mock-transfected cells in adhesion on collagen IV, collagen I, and fibronectin-coated surfaces. Although deleting individual extracellular domains of RAGE V, C1, and C2 affected cell adhesion to the ECM, the presence of the cytoplasmic domain was found to be necessary for signaling-dependent adhesion modulation for RAGE-mediated cell adhesion and cell spreading to the ECM. The FL- and TmCyto- RAGE variants exhibited comparable adherence rates, indicating that the transmembrane and cytoplasmic domains of RAGE can function independently, without requiring the extracellular domains. The surface localization of the DN-, ∆C1-, and ∆C2-RAGE variants did not closely correlate with cell adhesion or cell spreading to the ECM.

Studies from our lab and others suggest that RAGE can modulate the expression of various cell adhesion molecules and collagen genes, signaling to cell migration and adhesion [44,45,46]. We also observed that cells expressing the FL- and TmCyto-RAGE variants preferentially adhered to a collagen IV-coated surface. Studies have reported that conditional overexpression of RAGE in alveolar epithelial cells decreases collagen IV, providing a possible explanation for the increased cell adhesion [47]. In our study, we also found significant adhesion to collagen I and fibronectin-coated surfaces. The cell spreading data for the FL- and TmCyto-RAGE variants showed polarized cell morphology on all tested ECM proteins, except Matrigel. This observation is consistent with other studies on RAGE, where authors have demonstrated that cells expressing FL-RAGE exhibit no observable change in adhesion to Matrigel [29,48].

Our results showed that RAGE expression in HEK293 cells changed the transcription levels of specific cell adhesion molecule genes. RAGE HEK293 samples exhibited increased expression of integrin alpha 8 (ITGA8) and contactin1 (CNTN1) at both mRNA and protein levels. ITGA8 forms a functional heterodimer by associating with integrin beta 1 (ITGB1). The ITGB1 and ITGA8 heterodimer was reported to bind to the RGD sites of extracellular matrix molecules, such as fibronectin and vitronectin [49,50]. However, the data from our qPCR analysis showed no increase in the transcription for ITGB1 in RAGE HEK 293 samples. CNTN1 belongs to the neural adhesion molecule of the immunoglobulin (Ig) superfamily and associates with other cell surface proteins, such as tenascin-C, tenascin-R, and receptor protein tyrosine phosphatase β (RPTPβ), to initiate signaling [51]. Both CNTNI and ITGA8 genes were reported to have essential roles in mediating cell–cell and cell-extracellular matrix adhesion [49,52,53,54,55] and deregulation of both ITGA8 and CNTN1 has been suggested to play a crucial role in the EMT transition and metastasis of cancer cells by altering the expression of EMT-associated markers, such as Snail, E-cadherin, and *N*-cadherin [56,57,58]. Interestingly, RAGE was also reported to induce EMT transition, and the results from our data suggest that RAGE may cross talk with ITGA8 and CNTN1 to modulate oncogenic signaling [44,59].

We also observed decreased gene expression in melanoma cell adhesion molecule (MCAM), fibronectin-1 (FN1), and thrombospondin-1 (THBS1) in RAGE HEK293 cells. Until now, no study has demonstrated a direct relationship between the expression of these genes and the context of RAGE signaling. Few studies reported that RAGE shared structural homology with MCAM. Furthermore, RAGE and MCAM have also been reported to bind to S100A8/A9 protein heterodimers, mediating signaling in malignant melanoma. [29,60]. FN1 and THBS1 have been reported to have an essential role in tissue repair and wound healing response [61,62].

Global proteome analysis revealed that RAGE expression in HEK293 cells significantly upregulated the protein levels of the following adhesion molecules: NCAM, ITGA2, DAG1, and ITGA1. However, our qPCR analysis of the same genes showed no significant upregulation in transcriptional levels compared to WT HEK293. A possible explanation is that transcript levels do not always correlate with protein levels, as expression levels are also influenced by translation and degradation rates [63]. Consistent with the upregulation of adhesion-relevant surface proteins, RAGE also induced the expression of genes that were absent in WT HEK293 cells. The data suggest that RAGE can modulate the translational levels of these genes, although their transcriptional levels remain unchanged. Future studies will investigate the mechanistic and signaling correlation between RAGE and these proteins in more detail.

Among the identified proteins in RAGE HEK293 cells, only ITGA8 protein levels matched those of its mRNA, and the result was also supported by Western blot analysis. Other identified proteins, such as FLNA, CD44, CD99, and NPTN, were observed to have mRNA expression in WT HEK293 cells in our qPCR analysis (Supplementary Table S4); however, they were not detected at protein levels. To our surprise, we did not detect CNTN1 in the proteomics data, despite confirming its expression by Western blot. A possible explanation is the absence of surface localization, as these proteins are reported to form complexes with other surface proteins, such as Notch, which undergo regulated intramembrane proteolysis [58]. In the vasculature, RAGE expression was reported to stimulate VCAM1 and ICAM1 expression. From our data, we did not detect VCAM1 or ICAM1 at either the transcript or protein levels in RAGE HEK293 cells, suggesting that RAGE regulation of these two CAMs depends on the cell type [64]. We expected to observe increased expression of MCAM, FN1, and THBS1 based on our qPCR results in WT HEK293 cells; however, we were unable to detect these proteins in our proteomics study. However, WT HEK293 cells were found to express high levels of ephrin type A and B receptors, which were not found in RAGE HEK293 cells. Overall, our data suggest that RAGE expression selectively regulates the expression of specific cell-surface adhesion receptors in the tested conditions. Additional experimental studies will be necessary to elucidate the signaling pathways linking RAGE and specific cell adhesion molecules.

We knocked down RAGE in both RAGE HEK293 and MIA PaCa-2 cells. RAGE HEK293 cells showed a slightly higher knockdown level of RAGE in protein levels (~60%) compared to MIA PaCa-2 cells (~30%), which could be due to differences in the transfection efficiency between these cell types [65]. RAGE knockdown reduced cell adhesion compared to the control/scrambled shRNA transfected cells. This finding aligns with the other results of our study, which showed that RAGE overexpression significantly enhanced cell adhesion.

qPCR and proteomics studies demonstrated that RAGE increased ITGA8 expression at both the transcript and protein levels. We investigated ITGA8 levels following RAGE knockdown in RAGE-expressing HEK293 cells and the pancreatic cancer cell line Mia PaCa2-2, which is reported to express endogenous RAGE [66]. RAGE knockdown reduced expression of ITGA8 in both RAGE HEK293 and Mia PaCa-2 cells, further supporting the link between RAGE and ITGA8 expression in these cells.

## 5. Conclusions

In conclusion, our study provides an in-depth investigation of the role of RAGE’s extracellular domains in cell adhesion. The results from cell adhesion and cell spreading data highlighted the importance of the intercellular cytoplasmic domain (ICD), and we demonstrated that the presence of extracellular domains of RAGE contributed, but was not strictly required, for RAGE to modulate cell-extracellular matrix interactions. A surprising observation from this study was that the ICD of RAGE, even when over-expressed, could function similarly to the full-length RAGE receptor. This demonstrates that RAGE signaling through its cytoplasmic domain is a required element for its cell-adhesion mediating properties. The effects of RAGE on global changes in the expression of adhesion-relevant surface proteins were investigated, and ITGA8 was found to be significantly upregulated at both the mRNA and protein levels. Additionally, genes such as MCAM, FN1, and THBS1 were downregulated upon RAGE expression. The cell surface proteomics study was used as unbiased approach and identified additional cell adhesion molecules that were upregulated upon RAGE expression. NCAM, ITGA1, and ITGA2 showed greater than 3-fold changes. Additional studies will be necessary to further investigate the between RAGE and those proteins. Lastly, RAGE knockdown in RAGE HEK293 and MIA PaCa2 cells further confirmed the functional relationship between RAGE and ITGA8 expression. Effects of RAGE expression on cell adhesion, spreading, and ITGA8 expression were reversed by RAGE knockdown using shRNA.

Collectively, our study points to an alternative RAGE signaling mechanism that is most reliant on the cytoplasmic domain to modulate adhesion by regulating the expression of cell adhesion molecules, particularly ITGA8. It highlights the multifaceted role of RAGE in cell adhesion and gene regulation, opening new avenues for investigating RAGE’s functions in health and disease.

## Figures and Tables

**Table 1:** Summary of flow cytometry data showing increased FL1-A fluorescence intensity in RAGE variants compared to mock controls under both permeabilized and non-permeabilized conditions across three independent experiments. Results from three individual experiments are displayed as mean ± SD; n.d., not determined.

RAGE Variants	% Cells with Increased FL1-A Signal Compared to Mock in Non-Permeabilized Condition (FNP)	% Cells with Increased FL1-A Signal Compared to Mock in Permeabilized Condition (FP)	% Localized to the Cell Surface (FNP/FP) × 100
FL-RAGE	70 ± 5	80 ± 6	≈85
DN-RAGE	68 ± 7	95 ± 3	≈78
ΔC1-RAGE	54 ± 8	68 ± 4	≈75
ΔC2-RAGE	62 ± 4	70 ± 7	≈80
ΔV-RAGE	n.d.	80 ± 2	n.d.
TmCyto-RAGE	n.d.	81 ± 3	n.d.

## Data Availability

The original contributions presented in this study are included in the article/Supplementary Material. Further inquiries can be directed to the corresponding author.

## Supplementary Materials

The following supporting information can be downloaded at: https://www.mdpi.com/article/10.3390/cells14221805/s1, Figure S1. Extracted total RNA sample ran on a agarose gel. 200 ng of total RNA samples from WT HEK 293 and RAGE HEK 293 were run alongside a 100 bp ladder (GoldBio.com D001-500) on a 1.5% agarose gel containing 0.01% ethidium bromide. The 18S and 28S ribosomal RNA bands are visible and intact in these samples. The ratios of 260/280 nm for these extracted RNA samples were found to be ~1.8; Figure S2. Additional western blot data from different experiments representing Figure 2 of the main text. Protein expression in WT HEK 293 cells transfected with FL-RAGE and other RAGE variants at time points (A) 24, 48, and 72 h. It was observed that, over time, protein production significantly reduced, suggesting that the receptor undergoes continuous proteolytic cleavage. Actin was used as the loading control. The N-terminus antiRAGE 9A11 antibody was used to determine expression in FL-RAGE and DN-RAGE. For all other RAGE domain deletion variants, including FL-RAGE, expression was detected using the C-terminus antiRAGE D1A12 antibody; Figure S3. Additional images from different experiments representing Figure 3 of the main text. Confocal microscopy images of cells expressing the FL-RAGE and different domain deletion variants. For the DN-RAGE construct, the antiRAGE 9A11 antibody was used, and for all other constructs, the antiRAGE D1A12 antibody was used. The images were taken at 40X with an Olympus FV3000 at the same exposure time; Figure S4. Venn diagram representation of adhesion-relevant proteins identified by proteomics analysis (A) Venn diagrams showing the overlap of proteins identified in WT HEK293 and RAGE HEK293 samples across three independent experiments (Set I, Set II, and Set III); (B & C) Venn diagrams comparing the sets of proteins consistently identified within RAGE HEK293 and WT HEK293 samples across the three experiments (Set I, Set II, and Set III); Table S1. Nucleotide and protein sequence of RAGE mutants, Protein sequence of RAGE mutants; Table S2. Details of the primary antibodies used in this study, along with dilution specifications for western blotting (WB), immunofluorescence (IF), and flow-cytometry (FC) experiments; Table S3. Details of the gene specific primers used in this study; Table S4. Basal expression levels of genes mediating cell adhesion in WT HEK 293 and RAGE HEK 293 samples.

## Abbreviations

The following abbreviations are used in this manuscript:

RAGE	Receptor for Advanced Glycation End-products
FL-RAGE	Full-length RAGE construct
TmCyto-RAGE	Transmembrane cytoplasmic RAGE
DN-RAGE	Dominant negative RAGE
ΔV	Deleted V-domain RAGE Variant
ΔC1	Deleted C1-domain RAGE Variant
ΔC2	Deleted C2-domain RAGE Variant
ICD	Intercellular cytoplasmic domain
RTCA	Real Time Cell Analyzer
WT HEK 293	Wild Type Human Embryonic Kidney Cells
RAGE HEK293	HEK293 cells stably expressing RAGE
ALCAM/CD166	Activated leukocyte cell adhesion molecule
BCAM	Basal cell adhesion molecule
ITGA8	Integrin alpha 8
CNTN1	Contactin 1
THBS-1	Thrombospondin-1
FN1	Fibronectin 1
EPHA	Ephrin Type Receptor A
EPHB	Ephrin Type Receptor B
NCAM	Neural cell adhesion molecule
BSG	Basigin
NPTN	Neuroplastin
PODXL2	Podocalyxin-like protein 2
DAG1	Dystroglycan
MCAM	Melanoma cell adhesion molecule
EFNB1	Ephrin-B1
PTK7	Inactive tyrosine-protein kinase 7
FLNA	Filamin-A
ITGA1	Integrin alpha 1
ITGA2	Integrin alpha 2
ITGAV	Integrin alpha V
ITGB1	Integrin beta 1
CADH2	Cadherin-2
PLBX2	Plexin-B2
CD47	Leukocyte surface antigen CD47
TPBG	Trophoblast glycoprotein
