# Supplementary material for: The Receptor for Advanced Glycation End-Products (RAGE) Regulates Cell Adhesion Through Upregulation of ITGA8
Source: Cells. 2025 Nov 17;14(22):1805. doi: 10.3390/cells14221805 (PMC12650875; doi:10.3390/cells14221805)
Supplement: Supplementary file 1 [file cells-14-01805-s001.zip › cells-3844233-supplementary.pdf]

Supplementary Materials

Table S1. Nucleotide and protein sequence of RAGE mutants

Nucleotide sequence of rage mutants

FL-RAGE nucleotide sequence

ATGGCGGCGGGCACCGCGGTGGGCGCGTGGGTGCTGGTGCTGAGCCTGTGGGGCGCGGTGGTGGGCGCGCAG
AACATTACCGCGCGCATTGGCGAACCGCTGGTGCTGAAATGCAAAGGCGCGCCGAAAAAACCGCCGCAGCGC
CTGGAATGGAAACTGAACACCGGCCGCACCGAAGCGTGGAAAGTGCTGAGCCCGCAGGGCGGCGGCCCGTG
GGATAGCGTGGCGCGCGTGCTGCCGAACGGCAGCCTGTTTCTGCCGGCGGTGGGCATTCAGGATGAAGGCATT
TTTCGCTGCCAGGCGATGAACCGCAACGGCAAAGAAACCAAAAGCAACTATCGCGTGCGCGTGTATCAGATT
CCGGGCAAACCGGAAATTGTGGATAGCGCGAGCGAACTGACCGCGGGCGTGCCGAACAAAGTGGGCACCTG
CGTGAGCGAAGGCAGCTATCCGGCGGGCACCCTGAGCTGGCATCTGGATGGCAAACCGCTGGTGCCGAACGA
AAAAGGCGTGAGCGTGAAAGAACAGACCCGCCGCCATCCGGAAACCGGCCTGTTTACCCTGCAGAGCGAACT
GATGGTGACCCCGGCGCGCGGCGGCGATCCGCGCCCGACCTTTAGCTGCAGCTTTAGCCCGGGCCTGCCGCGC
CATCGCGCGCTGCGCACCGCGCCGATTCAGCCGCGCGTGTGGGAACCGGTGCCGCTGGAAGAAGTGCAGCTG
GTGGTGGAACCGGAAGGCGGCGCGGTGGCGCCGGGCGGCACCGTGACCCTGACCTGCGAAGTGCCGGCGCA
GCCGAGCCCGCAGATTCATTGGATGAAAGATGGCGTGCCGCTGCCGCTGCCGCCGAGCCCGGTGCTGATTCTG
CCGGAAATTGGCCCGCAGGATCAGGGCACCTATAGCTGCGTGGCGACCCATAGCAGCCATGGCCCGCAGGAA
AGCCGCGCGGTGAGCATTAGCATTATTGAACCGGGCGAAGAAGGCCCGACCGCGGGCAGCGTGGGCGGCAG
CGGCCTGGGCACCCTGGCGCTGGCGCTGGGCATTCTGGGCGGCCTGGGCACCGCGGCGCTGCTGATTGGCGTG
ATTCTGTGGCAGCGCCGCCAGCGCCGCGGCGAAGAACGCAAAGCGCCGGAAAACCAGGAAGAAGAAGAAG
AACGCGCGGAACTGAACCAGAGCGAAGAACCGGAAGCGGGCGAAAGCAGCACCGGCGGCCCG

ΔV-RAGE nucleotide sequence

ATGGCGGCGGGCACCGCGGTGGGCGCGTGGGTGCTGGTGCTGAGCCTGTGGGGCGCGGTGGTGGGCGCGGTG
TATCAGATTCCGGGCAAACCGGAAATTGTGGATAGCGCGAGCGAACTGACCGCGGGCGTGCCGAACAAAGTG
GGCACCTGCGTGAGCGAAGGCAGCTATCCGGCGGGCACCCTGAGCTGGCATCTGGATGGCAAACCGCTGGTG
CCGAACGAAAAAGGCGTGAGCGTGAAAGAACAGACCCGCCGCCATCCGGAAACCGGCCTGTTTACCCTGCA
GAGCGAACTGATGGTGACCCCGGCGCGCGGCGGCGATCCGCGCCCGACCTTTAGCTGCAGCTTTAGCCCGGGC
CTGCCGCGCCATCGCGCGCTGCGCACCGCGCCGATTCAGCCGCGCGTGTGGGAACCGGTGCCGCTGGAAGAA
GTGCAGCTGGTGGTGGAACCGGAAGGCGGCGCGGTGGCGCCGGGCGGCACCGTGACCCTGACCTGCGAAGTG
CCGGCGCAGCCGAGCCCGCAGATTCATTGGATGAAAGATGGCGTGCCGCTGCCGCTGCCGCCGAGCCCGGTG
CTGATTCTGCCGGAAATTGGCCCGCAGGATCAGGGCACCTATAGCTGCGTGGCGACCCATAGCAGCCATGGCC
CGCAGGAAAGCCGCGCGGTGAGCATTAGCATTATTGAACCGGGCGAAGAAGGCCCGACCGCGGGCAGCGTG
GGCGGCAGCGGCCTGGGCACCCTGGCGCTGGCGCTGGGCATTCTGGGCGGCCTGGGCACCGCGGCGCTGCTG
ATTGGCGTGATTCTGTGGCAGCGCCGCCAGCGCCGCGGCGAAGAACGCAAAGCGCCGGAAAACCAGGAAGA
AGAAGAAGAACGCGCGGAACTGAACCAGAGCGAAGAACCGGAAGCGGGCGAAAGCAGCACCGGCGGCCCG

ΔC1-RAGE nucleotide sequence

ATGGCGGCGGGCACCGCGGTGGGCGCGTGGGTGCTGGTGCTGAGCCTGTGGGGCGCGGTGGTGGGCGCGCAG
AACATTACCGCGCGCATTGGCGAACCGCTGGTGCTGAAATGCAAAGGCGCGCCGAAAAAACCGCCGCAGCGC
CTGGAATGGAAACTGAACACCGGCCGCACCGAAGCGTGGAAAGTGCTGAGCCCGCAGGGCGGCGGCCCGTG
GGATAGCGTGGCGCGCGTGCTGCCGAACGGCAGCCTGTTTCTGCCGGCGGTGGGCATTCAGGATGAAGGCATT
TTTCGCTGCCAGGCGATGAACCGCAACGGCAAAGAAACCAAAAGCAACTATCGCGTGCGCGTGTATCAGATT
CCGGGCAAAACCGCGCCGATTCAGCCGCGCGTGTGGGAACCGGTGCCGCTGGAAGAAGTGCAGCTGGTGGTG
GAACCGGAAGGCGGCGCGGTGGCGCCGGGCGGCACCGTGACCCTGACCTGCGAAGTGCCGGCGCAGCCGAG
CCCGCAGATTCATTGGATGAAAGATGGCGTGCCGCTGCCGCTGCCGCCGAGCCCGGTGCTGATTCTGCCGGAA
ATTGGCCCGCAGGATCAGGGCACCTATAGCTGCGTGGCGACCCATAGCAGCCATGGCCCGCAGGAAAGCCGC
GCGGTGAGCATTAGCATTATTGAACCGGGCGAAGAAGGCCCGACCGCGGGCAGCGTGGGCGGCAGCGGCCTG
GGCACCCTGGCGCTGGCGCTGGGCATTCTGGGCGGCCTGGGCACCGCGGCGCTGCTGATTGGCGTGATTCTGT
GGCAGCGCCGCCAGCGCCGCGGCGAAGAACGCAAAGCGCCGGAAAACCAGGAAGAAGAAGAAGAACGCGC
GGAACTGAACCAGAGCGAAGAACCGGAAGCGGGCGAAAGCAGCACCGGCGGCCCG

ΔC2-RAGE nucleotide sequence

ATGGCGGCGGGCACCGCGGTGGGCGCGTGGGTGCTGGTGCTGAGCCTGTGGGGCGCGGTGGTGGGCGCGCAGA
ACATTACCGCGCGCATTGGCGAACCGCTGGTGCTGAAATGCAAAGGCGCGCCGAAAAAACCGCCGCAGCGCCT
GGAATGGAAACTGAACACCGGCCGCACCGAAGCGTGGAAAGTGCTGAGCCCGCAGGGCGGCGGCCCGTGGGA
TAGCGTGGCGCGCGTGCTGCCGAACGGCAGCCTGTTTCTGCCGGCGGTGGGCATTCAGGATGAAGGCATTTTTCG
CTGCCAGGCGATGAACCGCAACGGCAAAGAAACCAAAAGCAACTATCGCGTGCGCGTGTATCAGATTCCGGGC
AAACCGGAAATTGTGGATAGCGCGAGCGAACTGACCGCGGGCGTGCCGAACAAAGTGGGCACCTGCGTGAGC
GAAGGCAGCTATCCGGCGGGCACCCTGAGCTGGCATCTGGATGGCAAACCGCTGGTGCCGAACGAAAAAGGCG
TGAGCGTGAAAGAACAGACCCGCCGCCATCCGGAAACCGGCCTGTTTACCCTGCAGAGCGAACTGATGGTGAC
CCCGGCGCGCGGCGGCGATCCGCGCCCGACCTTTAGCTGCAGCTTTAGCCCGGGCCTGCCGCGCCATCGCGCGC
TGCGCACCGCGCCGATTCAGCCGATTAGCATTATTGAACCGGGCGAAGAAGGCCCGACCGCGGGCAGCGTGGG
CGGCAGCGGCCTGGGCACCCTGGCGCTGGCGCTGGGCATTCTGGGCGGCCTGGGCACCGCGGCGCTGCTGATTG
GCGTGATTCTGTGGCAGCGCCGCCAGCGCCGCGGCGAAGAACGCAAAGCGCCGGAAAACCAGGAAGAAGAAG
AAGAACGCGCGGAACTGAACCAGAGCGAAGAACCGGAAGCGGGCGAAAGCAGCACCGGCGGCCCG

DN-RAGE nucleotide sequence

ATGGCGGCGGGCACCGCGGTGGGCGCGTGGGTGCTGGTGCTGAGCCTGTGGGGCGCGGTGGTGGGCGCGCAGA
ACATTACCGCGCGCATTGGCGAACCGCTGGTGCTGAAATGCAAAGGCGCGCCGAAAAAACCGCCGCAGCGCCT
GGAATGGAAACTGAACACCGGCCGCACCGAAGCGTGGAAAGTGCTGAGCCCGCAGGGCGGCGGCCCGTGGGA
TAGCGTGGCGCGCGTGCTGCCGAACGGCAGCCTGTTTCTGCCGGCGGTGGGCATTCAGGATGAAGGCATTTTTCG
CTGCCAGGCGATGAACCGCAACGGCAAAGAAACCAAAAGCAACTATCGCGTGCGCGTGTATCAGATTCCGGGC
AAACCGGAAATTGTGGATAGCGCGAGCGAACTGACCGCGGGCGTGCCGAACAAAGTGGGCACCTGCGTGAGC
GAAGGCAGCTATCCGGCGGGCACCCTGAGCTGGCATCTGGATGGCAAACCGCTGGTGCCGAACGAAAAAGGCG
TGAGCGTGAAAGAACAGACCCGCCGCCATCCGGAAACCGGCCTGTTTACCCTGCAGAGCGAACTGATGGTGAC
CCCGGCGCGCGGCGGCGATCCGCGCCCGACCTTTAGCTGCAGCTTTAGCCCGGGCCTGCCGCGCCATCGCGCGC
TGCGCACCGCGCCGATTCAGCCGCGCGTGTGGGAACCGGTGCCGCTGGAAGAAGTGCAGCTGGTGGTGGAACC
GGAAGGCGGCGCGGTGGCGCCGGGCGGCACCGTGACCCTGACCTGCGAAGTGCCGGCGCAGCCGAGCCCGCA
GATTCATTGGATGAAAGATGGCGTGCCGCTGCCGCTGCCGCCGAGCCCGGTGCTGATTCTGCCGGAAATTGGCC
CGCAGGATCAGGGCACCTATAGCTGCGTGGCGACCCATAGCAGCCATGGCCCGCAGGAAAGCCGCGCGGTGAG
CATTAGCATTATTGAACCGGGCGAAGAAGGCCCGACCGCGGGCAGCGTGGGCGGCAGCGGCCTGGGCACCCTG
GCGCTGGCGCTGGGCATTCTGGGCGGCCTGGGCACCGCGGCGCTGCTGATTGGCGTGATTCTGTGG

<u>Tm-Cyto nucleotide sequence</u>

ATGGCGGCGGGCACCGCGGTGGGCGCGTGGGTGCTGGTGCTGAGCCTGTGGGGCGCGGTGGTGGGCATTAGC
ATTATTGAACCGGGCGAAGAAGGCCCGACCGCGGGCAGCGTGGGCGGCAGCGGCCTGGGCACCCTGGCGCTG
GCGCTGGGCATTCTGGGCGGCCTGGGCACCGCGGCGCTGCTGATTGGCGTGATTCTGTGGCAGCGCCGCCAGC
GCCGCGGCGAAGAACGCAAAGCGCCGGAAAACCAGGAAGAAGAAGAAGAACGCGCGGAACTGAACCAGAG
CGAAGAACCGGAAGCGGGCGAAAGCAGCACCGGCGGCCCG

Protein sequence of RAGE mutants

<u>FL-RAGE protein sequence</u>

MAAGTAVGAWVLVLSLWGAVVGAQNITARIGEPLVLKCKGAPKKPPQRLEWKLNTGRTEAWKVLSPQGGGPWDS
VARVLPNGSLFLPAVGIQDEGIFRCQAMNRNGKETKSNYRVRVYQIPGKPEIVDSASELTAGVPNKVGTCVSEGSYPA
GTLSWHLDGKPLVPNEKGVSVKEQTRRHPETGLFTLQSELMVTPARGGDPRPTFSCSFSPGLPRHRALRTAPIQPRVW
EPVPLEEVQLVVEPEGGAVAPGGTVTLTCEVPAQPSPQIHWMKDGVPLPLPPSPVLILPEIGPQDQGTYSCVATHSSHG
PQESRAVSISIIEPGEEGPTAGSVGGSGLGTLALALGILGGLGTAALLIGVILWQRRQRRGEERKAPENQEEEEERAELN
QSEEPEAGESSTGGP

<u>ΔV-RAGE protein sequence</u>

MAAGTAVGAWVLVLSLWGAVVGAVYQIPGKPEIVDSASELTAGVPNKVGTCVSEGSYPAGTLSWHLDGKPLVPNE
KGVSVKEQTRRHPETGLFTLQSELMVTPARGGDPRPTFSCSFSPGLPRHRALRTAPIQPRVWEPVPLEEVQLVVEPEGG
AVAPGGTVTLTCEVPAQPSPQIHWMKDGVPLPLPPSPVLILPEIGPQDQGTYSCVATHSSHGPQESRAVSISIIEPGEEG
PTAGSVGGSGLGTLALALGILGGLGTAALLIGVILWQRRQRRGEERKAPENQEEEEERAELNQSEEPEAGESSTGGP

<u>ΔC1-RAGE protein sequence</u>

MAAGTAVGAWVLVLSLWGAVVGAQNITARIGEPLVLKCKGAPKKPPQRLEWKLNTGRTEAWKVLSPQGGGPWDS
VARVLPNGSLFLPAVGIQDEGIFRCQAMNRNGKETKSNYRVRVYQIPGKTAPIQPRVWEPVPLEEVQLVVEPEGGAV
APGGTVTLTCEVPAQPSPQIHWMKDGVPLPLPPSPVLILPEIGPQDQGTYSCVATHSSHGPQESRAVSISIIEPGEEGPTA
GSVGGSGLGTLALALGILGGLGTAALLIGVILWQRRQRRGEERKAPENQEEEEERAELNQSEEPEAGESSTGGP

<u>ΔC2-RAGE protein sequence</u>

MAAGTAVGAWVLVLSLWGAVVGAQNITARIGEPLVLKCKGAPKKPPQRLEWKLNTGRTEAWKVLSPQGGGPWDSV
ARVLPNGSLFLPAVGIQDEGIFRCQAMNRNGKETKSNYRVRVYQIPGKPEIVDSASELTAGVPNKVGTCVSEGSYPAGTL
SWHLDGKPLVPNEKGVSVKEQTRRHPETGLFTLQSELMVTPARGGDPRPTFSCSFSPGLPRHRALRTAPIQPISIIEPGEEG
PTAGSVGGSGLGTLALALGILGGLGTAALLIGVILWQRRQRRGEERKAPENQEEEEERAELNQSEEPEAGESSTGGP

<u>DN-RAGE protein sequence</u>

MAAGTAVGAWVLVLSLWGAVVGAQNITARIGEPLVLKCKGAPKKPPQRLEWKLNTGRTEAWKVLSPQGGGPWDS
VARVLPNGSLFLPAVGIQDEGIFRCQAMNRNGKETKSNYRVRVYQIPGKPEIVDSASELTAGVPNKVGTCVSEGSYPA
GTLSWHLDGKPLVPNEKGVSVKEQTRRHPETGLFTLQSELMVTPARGGDPRPTFSCSFSPGLPRHRALRTAPIQPRVW
EPVPLEEVQLVVEPEGGAVAPGGTVTLTCEVPAQPSPQIHWMKDGVPLPLPPSPVLILPEIGPQDQGTYSCVATHSSHG
PQESRAVSISIIEPGEEGPTAGSVGGSGLGTLALALGILGGLGTAALLIGVILW

<u>Tm-Cyto protein sequence</u>

MAAGTAVGAWVLVLSLWGAVVGISIIEPGEEGPTAGSVGGSGLGTLALALGILGGLGTAALLIGVILWQRRQRRGEER
KAPENQEEEEERAELNQSEEPEAGESSTGGP

Table S2. Details of the primary antibodies used in this study, along with dilution specifications for western blotting (WB), immunofluorescence (IF), and flow-cytometry (FC) experiments.

Antibody	Company	Catalog No.	Species	Dilution
RAGE (9A11)	Santa Cruz Biotechnology	Sc-80653	Mouse	1:1000 – WB<br>1:100 – IF and FC
RAGE (D1A12)	Cell Signaling Technology	6996	Rabbit	1:1000 – WB<br>1:100 – IF and FC
Human Integrin alpha 8 Antibody	R&D Systems	MAB6194	Mouse	1:2000 – WB<br>1:200 – FC
Human/Mouse/Rat Contactin-1 Antibody	R&D Systems	AF904	Goat	1:500 – WB
B-Actin	Cell Signaling Technology	4970	Rabbit	1:1000 – WB
HRP conjugated	Jackson Immuno Research	715-035-150	Anti-Mouse	1:500000 – WB
HRP conjugated	Jackson Immuno Research	711-035-152	Anti-Rabbit	1:500000 – WB
HRP conjugated	Jackson Immuno Research	705-035-147	Anti-Goat	1:500000 – WB
FITC conjugated	Jackson Immuno Research	115-095-062	Anti-Mouse	1:200 – IF and FC
FITC conjugated	Jackson Immuno Research	711-545-152	Anti-Rabbit	1:200 – IF and FC
Alexa Fluor® 488 Conjugate	Cell Signaling Technologies	4412	Anti-Rabbit	1:200 –FC
Alexa Fluor® 555 Conjugate	Cell Signaling Technologies	4413	Anti-Rabbit	1:200 –FC

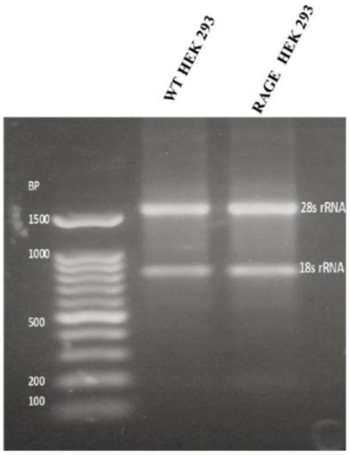

Figure S1: Extracted total RNA sample ran on a agarose gel. 200 ng of total RNA samples from WT HEK 293 and RAGE HEK 293 were run alongside a 100bp ladder (GoldBio.com D001-500) on a 1.5% agarose gel containing 0.01% ethidium bromide. The 18S and 28S ribosomal RNA bands are visible and intact in these samples. The ratios of 260/280 nm for these extracted RNA samples were found to be ~1.8.

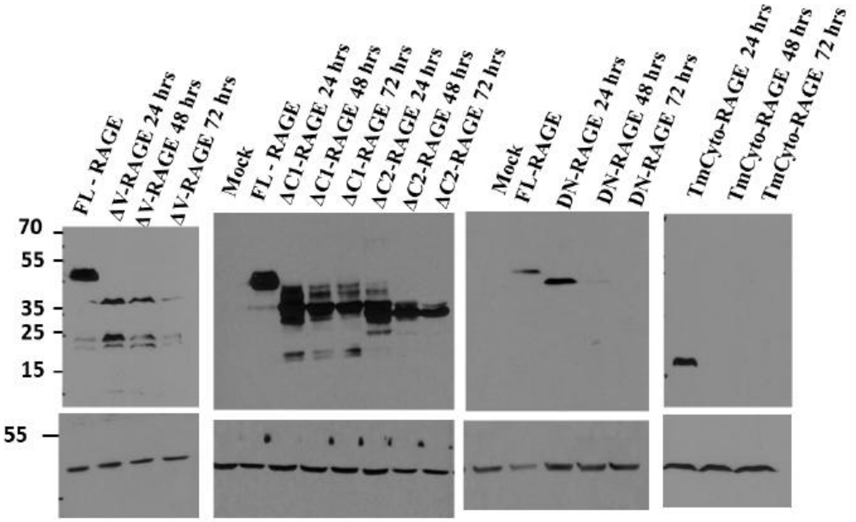

Figure S2: Additional western blot data from different experiments representing Figure 2 of the main text. Protein expression in WT HEK 293 cells transfected with FL-RAGE and other RAGE variants at time points (A) 24, 48, and 72 hours. It was observed that, over time, protein production significantly reduced, suggesting that the receptor undergoes continuous proteolytic cleavage. Actin was used as the loading control. The N-terminus antiRAGE 9A11 antibody was used to determine expression in FL-RAGE and DN-RAGE. For all other RAGE domain deletion variants, including FL-RAGE, expression was detected using the C-terminus antiRAGE D1A12 antibody.

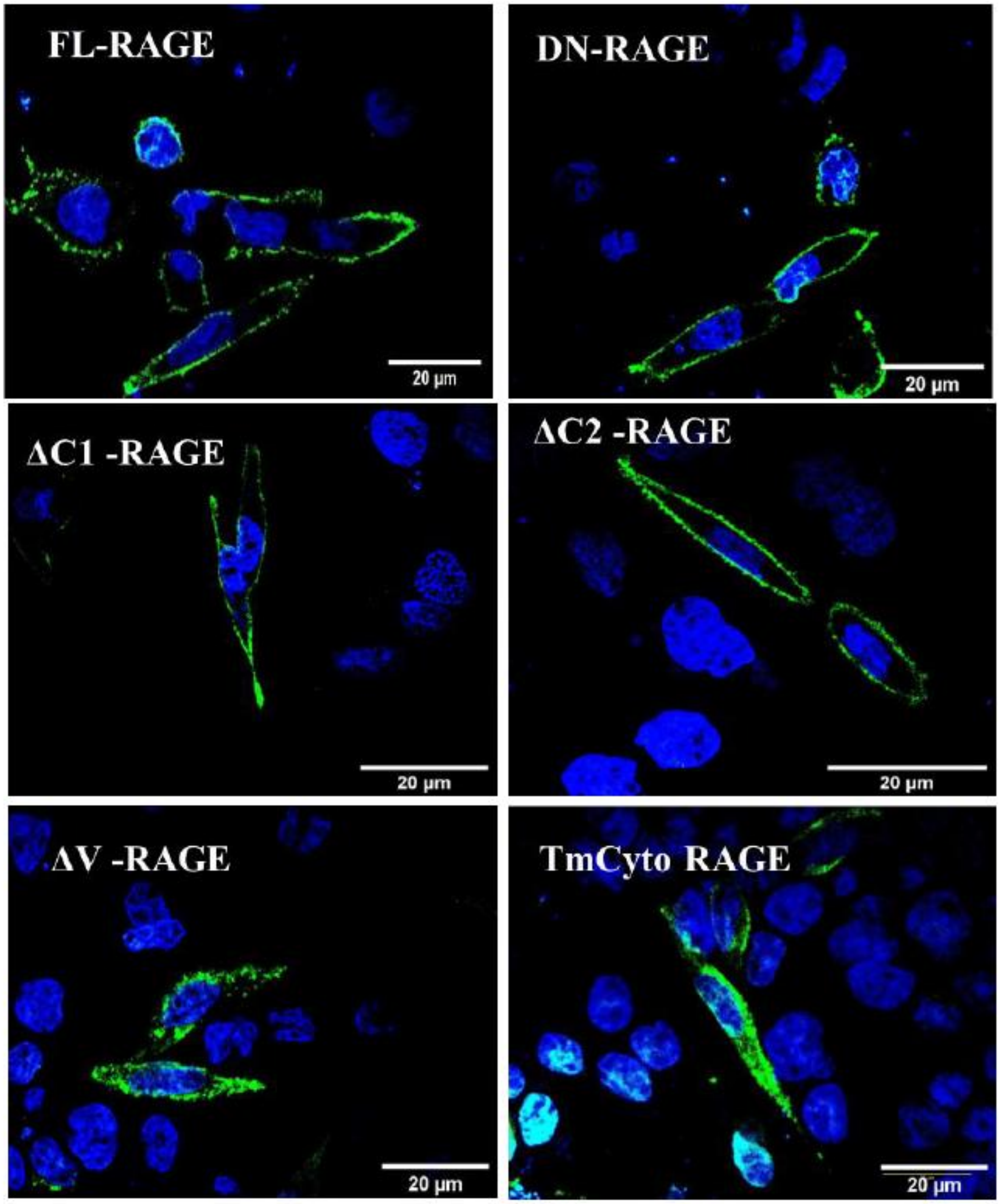

Figure S3: Additional images from different experiments representing Figure 3 of the main text. Confocal microscopy images of cells expressing the FL-RAGE and different domain deletion variants. For the DN-RAGE construct, the antiRAGE 9A11 antibody was used, and for all other constructs, the antiRAGE D1A12 antibody was used. The images were taken at 40X with an Olympus FV3000 at the same exposure time.

Table S3. Details of the gene specific primers used in this study.

Gene<br>(Gene ID)	Name	Primers (5′ → 3)
PGK1<br>NM_000291	Phosphoglycerate kinase	Forward: AGTCGGTAGTCCTTATGAGCC<br>Reverse: TTCCCAGAAGCATCTTTTCCC
ADAMTS13<br>NM_139026	ADAM metallopeptidase with thrombospondin type 1 motif	Forward: GGGTGCCCCAAATATCACAG<br>Reverse: CATCAGGCAACTCCAGGTCA
ALCAM<br>NM_001243281	Activated leukocyte cell adhesion molecule	Forward: ACTTGACGTACCTCAGAATCTCA<br>Reverse: CATCGTCGTACTGCACACTTT
CD133<br>NM_001145847	CD133 molecule (PROM1)	Forward: CAGAGTACAACGCCAAACCA<br>Reverse: AAATCACGATGAGGGTCAGC
CD24<br>NM_013230	CD24 molecule	Forward: CTGCAGTCAACAGCCAGTCT<br>Reverse: ACGTTTCTTGGCCTGAGTCT
CD36 NM_001001548	Platelet glycoprotein 4	Forward: CTTTGGCTTAATGAGACTGGGAC<br>Reverse: GCAACAAACATCACCACACCA
CD44<br>NM_000610	CD44 Molecule	Forward: TCCCTGCTACCACTTTGATG<br>Reverse: AGACGTACCAGCCATTTGTG
CDH1<br>NM_004360	Cadherin 1	Forward: CGAGAGCTACACGTTCACGG<br>Reverse: GGGTGTCGAGGGAAAAATAGG
CDH12<br>NM_004061	Cadherin 12	Forward: TTTGATGGAGGTCTCCTAACACC<br>Reverse: ACGTTTAACACGTTGGAAATGTG
CDH2<br>NM_021248	Cadherin 22	Forward: TGTATGTGGGCAAGATCCACT<br>Reverse: CTCGTCGATCAGGAAGATGGT
CNTN1<br>NM_175038	Contactin 1	Forward: CAGCCCTTTCCCGGTTTACAA<br>Reverse: TGCTTCTGACCATCCCGTAGT
Cola1h	Collagen type XVIII alpha 1 chain	Forward: GTTCCAGAGAATGCCGCTTG

NM_005261180		Reverse: CCCATCTGAGTCATCGCCTT
CTGF<br>NM_001901	Connective tissue growth factor	Forward: CAGCATGGACGTTCGTCTG<br>Reverse: AACCACGGTTTGGTCCTTGG
CTNNA1<br>NM_004903	Catenin (cadherin-associated protein), alpha 1	Forward: GGGGATAAAATTGCGAAGGAGA<br>Reverse: GTTGCCTCGCTTCACAGAAGA
CTNNB1<br>NM_001098209	Catenin beta 1	Forward: CATCTACACAGTTTGATGCTGCT<br>Reverse: GCAGTTTTGTCAGTTCAGGGA
CTNND1<br>NM_001085467	Catenin delta 1	Forward: GTGACAACACGGACAGTACAG<br>Reverse: TTCTTGCGGAAATCACGACCC
DDR1<br>NM_001202523	Discoidin domain receptor tyrosine kinase 1	Forward: AAGGGACATTTTGATCCTGCC<br>Reverse: CCTTGGGAAACACCGACCC
DDR2<br>NM_001014796	Discoidin domain receptor tyrosine kinase 2	Forward: CCAGTCAGTGGTCAGAGTCCA<br>Reverse: GGGTCCCCACCAGAGTGATAA
DESP/DSP<br>NM_004415	Desmoplakin	Forward: GCAGGATGTACTATTCTCGGC<br>Reverse: CCTGGATGGTGTTCTGGTTCT
ECAD<br>NM_004360	E-cadherin	Forward: GTCACTGACACCAACGATAATCCT<br>Reverse: TTTCAGTGTGGTGATTACGACGTTA
EPCAM<br>NM_002354	Epithelial cell adhesion molecule	Forward: TGATCCTGACTGCGATGAGAG<br>Reverse: CTTGTCTGTTCTTCTGACCCC
EZR<br>NM_003379	Ezrin	Forward: AGCGGCTGATCCCTCAAAG<br>Reverse: GGCATCAACTCCAAGCCAAAG
FN1<br>NM_212482	Fibronectin	Forward: CGGTGGCTGTCAGTCAAAG<br>Reverse: AAACCTCGGCTTCCTCCATAA
HAS3 NM_005329	Hyaluronan synthase 3	Forward: ATTATCAAGGCCACCTACGC<br>Reverse: GGAATGAGGCCAATGAAGTT
ICAM1	Intercellular adhesion molecule 1	Forward: CCAAGTTGTTGGGCATAGAG

NM_000201		Reverse: AGTCCAGTACACGGTGAGGA
ITGA1<br>NM_181501.1	Integrin Subunit Alpha 1	Forward: GTGCTTATTGGTTCTCCGTTAGT<br>Reverse: CACAAGCCAGAAATCCTCCAT
ITGA2<br>NM_002203	Integrin Subunit Alpha 2	Forward: CCTACAATGTTGGTCTCCCAGA<br>Reverse: AGTAACCAGTTGCCTTTTGGATT
ITGA3<br>NM_005501	Integrin alpha 3	Forward: TGTGGCTTGGAGTGACTGTG<br>Reverse: TCATTGCCTCGCAGCTAGC
ITGA4<br>NM_000885	Integrin Subunit Alpha 4	Forward: AGCCCTAATGGAGAACCTTGT<br>Reverse: CCAGTGGGGAGCTTATTTTCAT
INTGA5<br>NM_002205	Integrin Subunit Alpha<br>5	Forward: ATCTGTGAGGTCGAAACAGGA<br>Reverse: TGGAGCATACTCAACAGTCTTTG
ITGA7<br>NM_001144997	Integrin alpha 7	Forward: CTGACTCCATGTTCGGGATCA<br>Reverse: CACCTGTGAAGGTTTGGCG
ITGA8<br>NM_0032638	Integrin, alpha 8	Forward: GAATGGAGACCTTATTGTGGGA<br>Reverse: GAGCCACTTCCGTCTGCTTT
ITGA10<br>NM_003637	Integrin alpha 10	Forward: ACTTAGGTGACTACCAACTGGG<br>Reverse: CCACAAGCACGAGACCAGA
ITGA11<br>NM_001004439	Integrin subunit alpha 11	Forward: GTCACCCTGTCCAACGTGTC<br>Reverse: ACATCCCTGTGGTGTAGTAGG
ITGAL<br>NM_001114380	Integrin, alpha L	Forward: TGCTTATCATCATCACGGATGG<br>Reverse: CTCTCCTTGGTCTGAAAATGCT
ITGAM<br>NM_000632	Integrin alpha M	Forward: CACATGACTTTCGGCGGATGA<br>Reverse: GCTGCGTTATTGGCTTCACC
ITGAM<br>NM_001145808	Integrin alpha M	Forward: ACTGGTGAAGCCAATAACGCA<br>Reverse: TCCGTGATGACAACTAGGATCTT
ITGAV	Integrin alpha V	Forward: ATCTGTGAGGTCGAAACAGGA

NM_001145000		Reverse: TGGAGCATACTCAACAGTCTTTG
ITGB1<br>NM_002211	Integrin beta 1	Forward: CCTACTTCTGCACGATGTGATG<br>Reverse: CCTTTGCTACGGTTGGTTACATT
ITGB2<br>NM_000211	Integrin beta 2 chain	Forward: AGTGTGACACCATCAACTGTG<br>Reverse: GCACTCGCATACGTTGCAG
ITGB2<br>NM_000211	Integrin, beta 2	Forward: TGCGTCCTCTCTCAGGAGTG<br>Reverse: GGTCCATGATGTCGTCAGCC
ITGB3<br>NM_000212	Integrin beta 3	Forward: GTGACCTGAAGGAGAATCTGC<br>Reverse: CCGGAGTGCAATCCTCTGG
ITGB4<br>NM_001005619	Integrin beta 4	Forward: CTCCACCGAGTCAGCCTTC<br>Reverse: CGGGTAGTCCTGTGTCCTGTA
ITGB5<br>NM_002213	Integrin beta 5	Forward: GGAAGTTCGGAAACAGAGGGT<br>Reverse: CTTTCGCCAGCCAATCTTCTC
MCAM<br>NM_006500	Melanoma cell adhesion molecule	Forward: AGCTCCGCGTCTACAAAGC<br>Reverse: CTACACAGGTAGCGACCTCC
MPZL1<br>NM_001146191	Myelin protein zero like 1	Forward: ACGCCAAAAGAAATCTTCGTGG<br>Reverse: TCAACCCGCCAGTCGTACTA
MSN<br>NM_002444	Meosin	Forward: ATGCCCAAAACGATCAGTGTG<br>Reverse: ACTTGGCACGGAACTTAAAGAG
NCAM1<br>NM_001076682	Neural cell adhesion molecule	Forward: GGCATTTACAAGTGTGTGGTTAC<br>Reverse: TTGGCGCATTCTTGAACATGA
NPTN<br>NM_017455	Neuroplastin	Forward: GAGGTCATTATTCGAGACAGCC<br>Reverse: TTGATCCTGTACTCCATGTTGC
PCDHA3<br>NM_031497	Protocadherin alpha 3	Forward: GTTTTCGCTAGAGGGCGCAT<br>Reverse: CAACACGAGTCCAAGGGATTTA

PECAM1<br>NM_000442	Platelet and endothelial cell adhesion molecule	Forward: AACAGTGTTGACATGAAGAGCC<br>Reverse: TGTAAAACAGCACGTCATCCTT
PLXB2<br>NM_012401	Plexin B2	Forward: AGCCTCTTCAAGGGCATCTG<br>Reverse: GCCACGAAAGACTTCTCCCC
PNN NM_002687.3	Desmosome-associated protein	Forward: GTCGCCGTGAGAACTTTGC<br>GGTCCTCCTCCACTATCTGAGA
RAGE<br>NM_001136	Receptor for advanced glycation endproducts	Forward: GGGCAGTAGTAGGTGCTCAAA<br>Reverse: CGGCCTGTGTTCAGTTTCCAT
RDX<br>NM_002906	Radixin	Forward: AATTGTGGCTAGGTGTTGATGC<br>Reverse: GGTGCCTTTTTGTCGATTGGC
SELE<br>NM_000450	Selectin E	Forward: CAGCAAAGGTACACACACCTG<br>Reverse: CAGACCCACACATTGTTGACTT
SELL<br>NM_000655	Selectin L	Forward: ACCCAGAGGGACTTATGGAAC<br>Reverse: GCAGAATCTTCTAGCCCTTTGC
SELP<br>NM_003005	Selectin P	Forward: ACTGCCAGAATCGCTACACAG<br>Reverse: CACCCATGTCCATGTCTTATTGT
SGCE<br>NM_001099400	Sarcoglycan epsilon	Forward: GGCGTTTATGTCATGGTTGGT<br>Reverse: AGGTGGACACTTGCTTTGTTT
THBS1<br>NM_003246	Thrombospondin-1	Forward: AGACTCCGCATCGCAAAGG<br>Reverse: TCACCACGTTGTTGTCAAGGG
THBS2<br>NM_003247	Thrombospondin-2	Forward: GACACGCTGGATCTCACCTAC<br>Reverse: GAAGCTGTCTATGAGGTCGCA
THBS3<br>NM_001252607	Thrombospondin 3	Forward: ATGGAGACGCAGGAACTTCG<br>Reverse: AGCTACCATCTGCCGAGACT
VCAM1<br>NM_001078	Vascular cell adhesion molecule 1	Forward: CAGATAGACAGCCCTCTGAGC<br>Reverse: CTCCACCTGGATTCCCTTT

VCAN<br>NM_004385	Versican core protein	Forward: GTAACCCATGCGCTACATAAAGT<br>Reverse: GGCAAAGTAGGCATCGTTGAAA
VIM<br>NM_003380	vimentin	Forward: GGCAGAAGAATGGTACAAATCC<br>Reverse: CTTCCAGCAGCTTCCTGTAG
VTN<br>NM_000638	Vitronectin	Forward: CGGGGATGTGTTCACTATGCC<br>Reverse: GTGTCTGCTCAGGATTCCCTT

Table S4. Basal expression levels of genes mediating cell adhesion in WT HEK 293 and RAGE HEK 293 samples

	**Gene (Gene ID)**	**WT HEK293 C_t values**	**RAGE HEK293 Ct values**
1	ADAMTS13<br>NM_139026	28.5±1.0	27.9±0.9
2	ALCAM<br>NM_001243281	24.7±1.5	24.7±1.0
3	CD133<br>NM_001145847	30.5±0.6	29.3±0.7
4	CD24<br>NM_013230	25.6±0.9	27.2±0.8
5	CD36<br>NM_001001548	36.4±1.7	37.1±1.5
6	CD44<br>NM_000610	30.1±1.6	30.2±0.5
7	CDH1<br>NM_004360	28.1±2.0	27.7±0.5
8	CDH2<br>NM_021248	32.6±2.6	32.3±0.6
9	CDH12<br>NM_004061	31.1±2.0	30.8±0.5
10	CNTN1<br>NM_175038	29.7±0.4	26.7±0.8
11	Cola1h<br>NM_005261180	29.5±0.9	31.1±0.6
12	CTGF<br>NM_001901	23.0±2.2	25.5±1.8
13	CTNNA1<br>NM_004903	22.1±1.4	22.6±0.8
14	CTNNB1<br>NM_001098209	25.3±3.9	24.1±2.4

15	CTNND1<br>NM_001085467	23.2±0.8	23.4±1.4
16	DDR1<br>NM_001202523	25.0±1.2	24.9±0.5
17	DDR2<br>NM_001014796	29.4±1.0	28.7±1.3
18	DESP/DSP<br>NM_004415	23.4±1.9	24.0±0.9
19	ECAD<br>NM_004360	27.2±1.2	27.8±0.8
20	EPCAM<br>NM_002354	22.0±1.5	23.1±1.0
21	EZR<br>NM_003379	22.6±1.0	23.4±0.8
22	FN1<br>NM_212482	22.7±0.8	25.3±0.5
23	HAS3<br>NM_005329	27.1±0.6	27.5±0.6
24	ICAM1<br>NM_000201	30.2±1.8	29.2±1.0
25	ITGA1<br>NM_181501.1	28.0±2.1	29.0±1.0
26	ITGA2<br>NM_002203	29.8±2.1	30.1±1.0
27	ITGA3<br>NM_005501	31.3±2.6	31.4±0.9
28	ITGA4<br>NM_000885	27.1±1.5	27.3±1.1
29	ITGA5<br>NM_002205	29.5±2.2	30.4±1.1
30	ITGA7<br>NM_001144997	26.1±0.9	26.2±0.6
31	ITGA8<br>NM_0032638	29.3±0.7	25.8±0.7
32	ITGA10<br>NM_003637	29.3±2.1	29.5±0.7
33	ITGA11<br>NM_001004439	25.4±1.9	26.4±2.5
34	ITGAL<br>NM_001114380	30.8±2.7	30.5±1.0
35	ITGAM<br>NM_000632	30.8±3.2	30.2±1.2
36	ITGAM<br>NM_001145808	32.6±2.2	33.0±0.7

37	ITGAV<br>NM_001145000	25.0±0.7	26.4±0.5
38	ITGB1<br>NM_002211	27.0±0.8	27.8±0.6
39	ITGB2<br>NM_000211	32.2±3.4	33.0±1.6
40	ITGB2<br>NM_000211	31.5±1.0	31.3±2.7
41	ITGB3<br>NM_000212	26.4±0.9	27.8±0.5
42	ITGB4<br>NM_001005619	25.9±1.4	26.4±0.8
43	ITGB5<br>NM_002213	25.4±0.8	25.7±0.6
44	MCAM<br>NM_006500	26.9±1.1	29.5±0.7
45	MPZL1<br>NM_001146191	23.5±1.3	24.0±0.9
46	MSN<br>NM_002444	23.4±0.7	23.4±0.4
47	NCAM1<br>NM_001076682	27.8±4.2	28.7±4.0
48	NPTN<br>NM_017455	22.9±1.3	23.5±1.0
49	PCDHA3<br>NM_031497	31.9±2.8	31.2±1.1
50	PECAM1<br>NM_000442	26.3±1.1	27.0±1.3
51	PLXB2<br>NM_012401	27.6±1.9	27.4±0.5
52	PNN<br>NM_002687.3	25.3±0.3	26.0±1.7
53	RAGE<br>NM_001136	28.1±1.8	19.0±1.4
54	RDX<br>NM_002906	21.5±0.6	22.5±0.4
55	SELE<br>NM_000450	29.2±2.7	28.3±1.0
56	SELL<br>NM_000655	29.8±2.4	29.7±1.0
57	SELP<br>NM_003005	29.7±3.6	29.4±1.7
58	SGCE<br>NM_001099400	27.1±4.0	26.5±3.1

59	THBS1<br>NM_003246	25.4±0.8	28.9±0.5
60	THBS2<br>NM_003247	28.8±1.9	28.4±0.7
61	THBS3<br>NM_001252607	29.9±1.1	28.8±0.6
62	VCAM1<br>NM_001078	30.0±1.7	29.3±0.8
63	VCAN<br>NM_004385	25.0±0.5	24.9±0.6
64	VIM<br>NM_003380	24.3±1.2	25.2±0.5
65	VTN<br>NM_000638	27.0±1.5	26.2±1.1

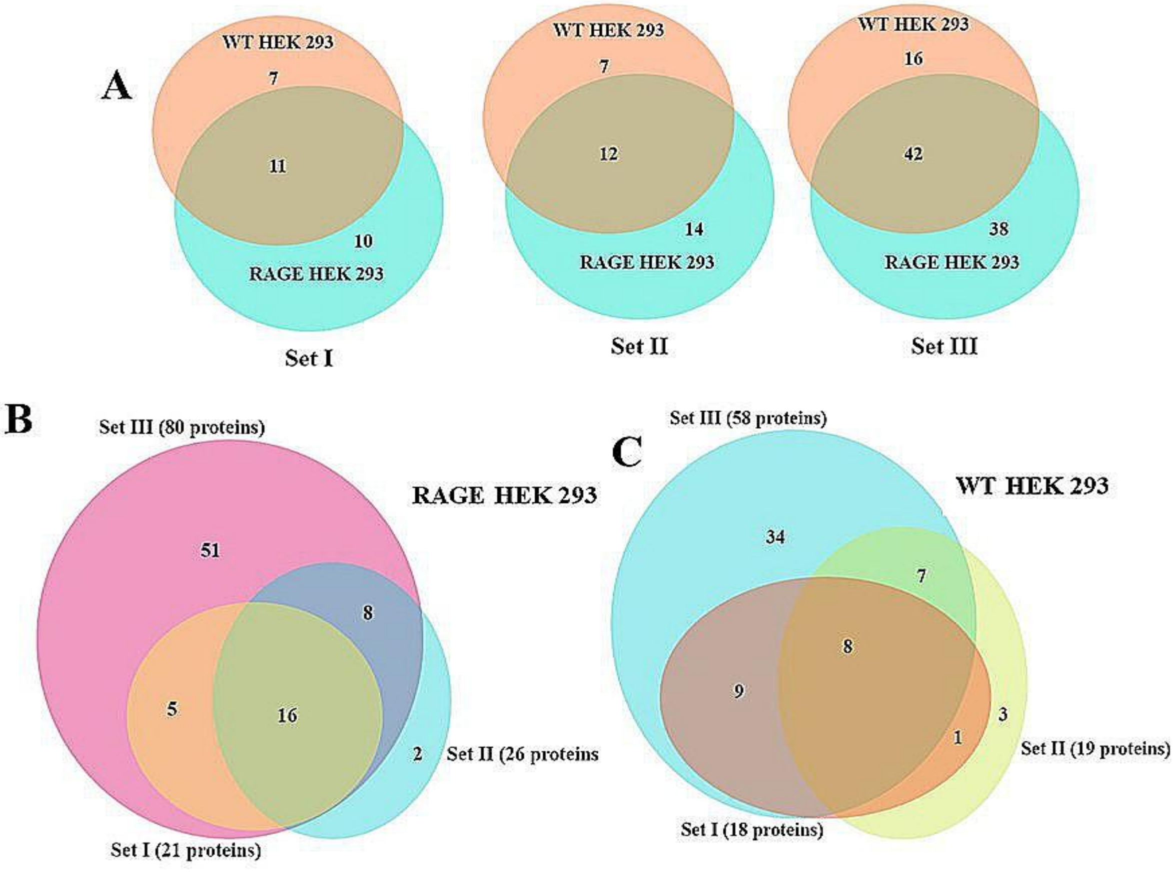

Figure S4. Venn diagram representation of adhesion-relevant proteins identified by proteomics analysis (A) Venn diagrams showing the overlap of proteins identified in WT HEK293 and RAGE HEK293 samples across three independent experiments (Set I, Set II, and Set III); (B & C) Venn diagrams comparing the sets of proteins consistently identified within RAGE HEK293 and WT HEK293 samples across the three experiments (Set I, Set II, and Set III).
